# Heat Transfer and Hydrodynamic Properties Using Different Metal-Oxide Nanostructures in Horizontal Concentric Annular Tube: An Optimization Study

**DOI:** 10.3390/nano11081979

**Published:** 2021-07-31

**Authors:** Omer A. Alawi, Ali H. Abdelrazek, Mohammed Suleman Aldlemy, Waqar Ahmed, Omar A. Hussein, Sukaina Tuama Ghafel, Khaled Mohamed Khedher, Miklas Scholz, Zaher Mundher Yaseen

**Affiliations:** 1 Department of Thermofluids, School of Mechanical Engineering, Universiti Teknologi Malaysia, Skudai 81310, Malaysia; omeralawi@utm.my; 2 Department of Mechanical Engineering, University of Malaya, Kuala Lumpur 50603, Malaysia; ali_hassan80@siswa.um.edu.my; 3 Department of Mechanical Engineering, Collage of Mechanical Engineering Technology, Benghazi, Libya; maldlemy@ceb.edu.ly; 4 Malaysia-Japan International Institute of Technology (MJIIT), Kuala Lumpur 54100, Malaysia; waqarum.ah@gmail.com; 5 Institute for Advanced Studies, University of Malaya, Kuala Lumpur 50603, Malaysia; 6 Department of Mechanical Engineering, College of Engineering-Alsharkat, Tikrit University, Tikrit 34005, Iraq; omar-assi81@tu.edu.iq; 7 Optical Department, Al-Ayen University, Nasiriyah 64001, Iraq; tuamasukaina88@alayen.edu.iq; 8 Department of Civil Engineering, College of Engineering, King Khalid University, Abha 61421, Saudi Arabia; kkhedher@kku.edu.sa; 9 Department of Civil Engineering, High Institute of Technological Studies, Mrezgua University Campus, Nabeul 8000, Tunisia; 10 Division of Water Resources Engineering, Faculty of Engineering, Lund University, P.O. Box 118, 221 00 Lund, Sweden; 11 Department of Civil Engineering Science, School of Civil Engineering and the Built Environment, University of Johannesburg, Kingsway Campus, P.O. Box 524, Aukland Park 2006, Johannesburg 2092, South Africa; 12 Department of Town Planning, Engineering Networks and Systems, South Ural State University (National Research University), 76, Lenin prospekt, 454080 Chelyabinsk, Russia; 13 Institute of Environmental Engineering, Wroclaw University of Environmental and Life Sciences, ul. Norwida 25, 50-375 Wrocław, Poland; 14 New era and Development in Civil Engineering Research Group, Scientific Research Center, Al-Ayen University, Thi-Qar 64001, Iraq; 15 College of Creative Design, Asia University, Taichung City, Taiwan

**Keywords:** concentric annuli, turbulent mixed convection, nanofluids, nanoparticle shape, hydrodynamic properties

## Abstract

Numerical studies were performed to estimate the heat transfer and hydrodynamic properties of a forced convection turbulent flow using three-dimensional horizontal concentric annuli. This paper applied the standard k–ε turbulence model for the flow range 1 × 10^4^ ≤ Re ≥ 24 × 10^3^. A wide range of parameters like different nanomaterials (Al_2_O_3_, CuO, SiO_2_ and ZnO), different particle nanoshapes (spherical, cylindrical, blades, platelets and bricks), different heat flux ratio (HFR) (0, 0.5, 1 and 2) and different aspect ratios (AR) (1.5, 2, 2.5 and 3) were examined. Also, the effect of inner cylinder rotation was discussed. An experiment was conducted out using a field-emission scanning electron microscope (FE-SEM) to characterize metallic oxides in spherical morphologies. Nano-platelet particles showed the best enhancements in heat transfer properties, followed by nano-cylinders, nano-bricks, nano-blades, and nano-spheres. The maximum heat transfer enhancement was found in SiO_2_, followed by ZnO, CuO, and Al_2_O_3_, in that order. Meanwhile, the effect of the HFR parameter was insignificant. At Re = 24,000, the inner wall rotation enhanced the heat transfer about 47.94%, 43.03%, 42.06% and 39.79% for SiO_2_, ZnO, CuO and Al_2_O_3_, respectively. Moreover, the AR of 2.5 presented the higher heat transfer improvement followed by 3, 2, and 1.5.

## 1. Introduction

### 1.1. Research Background and Motivation

Heat exchangers are used extensively in thermal engineering applications. Specifically, annuli pipes are used in various applications such as electronic equipment, Heating, ventilation, air conditioning (HVAC), nuclear reactors, turbomachinery and gas turbines. In this regard, researchers and experts from all around the world are interested in developing new strategies to improve the heat transfer efficiency of annular tubes [1,2,3]. The primary heat transport fluids like pure water (DW), engine oil (EO), and ethylene glycol (EG) show low thermal efficiency [4,5]. Therefore, many researchers have been working on improving the thermal-physical properties of heat transfer fluids for better enhancements in heat transfer and hydrodynamic properties [6,7,8]. Experimental, theoretical, and numerical efforts have been performed to suspend nanoparticles in host fluids to produce nanofluids as alternatives to the basic working fluids [9,10,11,12]. Adding particles, characterized by nano dimensions, is found to improve a number of properties of base conventional engineered material significantly [13], and therefore has been applied in a number of practical applications, for example porous media [14], fuel cells [15], the power industry [16], and medical science [17], etc.

### 1.2. Adopted Literature Review on Annulus Heat Transfer Enhancement

Over the past decade, a remarkable advancement in the domain of heat transfer technologies can be noticed. This is owing to the massive demand for the diverse applications of high heat flux reduction. However, the introduced fluids mentioned in the literature (e.g., oil, water, ethylene, etc.) are associated with the limitation of low conductivity in addition to the shortcomings to meet the high standard of heat transfer properties. As per the high demand of exploring new modern technologies, the development of new types of fluids that are characterized with an effective and efficient heat transfer exchange performance. For this purpose, several propositions have been suggested and incorporated such as including microelectronics or chemical production.

Heat transfer properties were tested in a horizontal concentric annulus using Cu, Ag, TiO_2,_ and Al_2_O_3_ suspended in DW with different volume concentrations [18]. Nanoparticles with excellent thermal conductivity significantly improve heat transfer properties for high Rayleigh numbers and high L/D ratios. On the other hand, nanoparticles with low thermal conductivity produce a reduction in heat transfer at intermediate values of the Rayleigh number. The inclusion of Al_2_O_3_ nanoparticles enhances heat transfer for Ra = 10^3^ and Ra = 10^5^. On the other hand, the addition of nanoparticles has only a minimal effect on heat transfer properties for Ra = 10^4^. Cu-H_2_O nanofluids were tested mathematically and numerically in a semi-annulus for natural convection [19]. Effect of Cu nanoparticles was more pronounced at low Ra than at high Ra due to greater enhancement, and increasing Ra showed a decrease in heat transfer enhancement ratio. Experimental studies on heat transfer and sub-cooled flow boiling using CuO-H_2_O nanofluids inside a vertical annulus were conducted in the range of (353 kg/m^2^ s–1059 kg/m^2^ s) [20]. The heat transfer rate was enhanced due to increasing the value of mass flux and CuO concentration in the force convective and nucleate boiling regions. In contrast, increasing the solid nanoparticle concentration did not affect the pressure drop. The lattice Boltzmann method (LBM) was applied to study Cu-H_2_O nanofluid in horizontal cylindrical annulus with inner triangular cylinder [21]. Their results showed that the heat transfer enhanced by increasing the CuO volume concentration and when the inner pipe moved downward. The two-phase flow was applied to solve the laminar Al_2_O_3_-H_2_O flow in differentially-heated horizontal annuli [22]. As Al_2_O_3_ nanoparticle size and radius of the inner cylinder decreased, the radius ratio, the temperature of the cooled cylinder and the temperature difference increased, the impact of nanoparticle dispersion on thermal performance increased. 3D geometry was modeled to examine the Al_2_O_3_-H_2_O two-phase flow in an annulus [23]. The computed findings demonstrated that increasing the volume percentage raised the Nu at the inner and outer walls for a given Re and Gr, but has no influence on the friction factor. In conclusion, Both Nu_avg_ and C_f_ showed higher values at the inner cylinder than the outer cylinder. Izadi et al., [2] used Al_2_O_3_–DW to examine an annulus’s hydrodynamics and thermal performances. In general, the heat transfer rate was enhanced by increasing the Al_2_O_3_ volume fraction. Also, high Nu_avg_ was observed at a peak mean nanoparticles concentration for Al_2_O_3_/H_2_O inside annuli, meanwhile, Nu_avg_ only increased insignificantly with the solid concentration of TiO_2_/H_2_O [24]. An annular pipe was tested to study the turbulent flow of different metal oxides (CuO, Al_2_O_3_, and SiO_2_) suspended in Pure DW and EG [25]. The Nu_avg_ increased by adding more nanoparticles to the base fluids. The effect of 0.002 vol.%- TiO_2_/DW and 0.02 vol.%- TiO_2_/DW nanofluids on the heat transfer pressure loss performance was studied inside a concentric horizontal tube with 8000 ≤ Re ≤ 51,000 [26]. The Nu_avg_ increased by increasing the Re and adding more nanoparticles to the host fluid. Furthermore, the Nu_avg_ values of nanofluids were higher compared to the host fluid. Three different mass fractions of MWCNTs were chosen for use in an annular heat exchanger from a green synthesis [12]. The thermo-physical parameters of the nanostructures were measured after they were characterized with various instruments. The convective heat transfer coefficient and Nusselt number were found to be 35.89% and 20.15%, respectively, for 0.175 wt.% and Re of 7944. Recently, hybrid nanofluids (Al_2_O_3_@MWCNTs)/H_2_O was prepared as working fluids in the annular passage of various eccentricities [27]. For the concentric case, the greatest enhancement of the convection heat transfer coefficient was 6.49%, 4.86%, and 2.98%, respectively, for concentrations of 0.1 wt.%, 0.075 wt.% percent, and 0.05 wt.%.

### 1.3. Research Motivation

It was observed from the reported previous studies that most of the conducted research was established using a single type of nanofluids or hybrids of different types of nanomaterials. This results in some limitations for the better understanding of the effect of nanoparticle shape on the heat transfer and hydrodynamic properties. Hence, the current research was motivated to be conducted on the investigation of a wide range of parameters such as different nanomaterials (Al_2_O_3_, CuO, SiO_2_ and ZnO), different particle nano-shapes (spherical, cylindrical, blades, platelets and bricks), different heat flux ratio (HFR) (0, 0.5, 1 and 2) and different aspect ratios (AR) (1.5, 2, 2.5 and 3). In addition, the effect of inner cylinder rotation was analyzed and discussed. In summary, the research findings can provide a general visualization of the various types of nanofluids/nano-shapes’ behavior.

### 1.4. Research Objectives

As per the mentioned literature, no previous research has investigated the heat transfer and hydrodynamic performance of a concentric pipe using different types of nanoparticles such as aluminum oxide, silicon dioxide, copper oxide and zinc oxide, the metal oxides in morphologies like (nanospherical, nanoblades, nanoplatelets, nanocylinders and nanobricks) [28]. An experimental study was conducted to veirfy the spherical morphologies using an electron microscope. Thermophysical properties of nanofluids in different shapes suspended in DW were estimated via correlations and equations for different volume concentrations at 293 K. Moreover, a geometry in 3-D was built and solved via computational fluid dynamics (CFD) using ANSYS-Fluid flow analysis to examine different testing settings for heat transfer and pressure loss optimizations. The fully-developed turbulent flow in the range of 1 × 10^4^ ≤ Re ≤ 24 × 10^3^ was selected to test the impacts of different heat flux ratios (HFR: 0, 0.5, 1, and 2) and different aspect ratios (AR: 1.5, 2, 2.5 and 3). Besides, the rotation of the inner cylinder was taken into account. The influences of different cases on the temperature and velocity of annuli were illustrated through contours presentations.

## 2. Thermophysical Properties of Nanofluids

Most earlier investigations used experimentally [29] to test the thermal-physical properties of nanofluids. Because the method is costly, several other numerical/mathematical methods with high accuracy are available, such as molecular dynamics (MD), classical density functional theory (c-DFT) and others [30,31]. In this study, the Brownian motion was included in the measurements of thermal conductivity and dynamic viscosity, meanwhile, the Pac and Cho [32] equations were used to estimate the density and specific heat capacity of nanofluids.

### 2.1. Nano-Spherical Particles

Thermal and physical properties of the effective nanofluids in different types and shapes were theoretically measured at various volume fractions in the range of (0–4%-vol.) under the condition of 293 K. Essentially, the required thermal-physical properties for the simulations are effective density (*ρ*_eff_), effective specific heat capacity (*Cp*_eff_), effective thermal conductivity (*k*_eff_), and effective dynamic viscosity (*μ*_eff_).

The effective thermal conductivity and effective dynamic viscosity of nanofluids can be obtained by taking into account the Brownian motions between solid particles in the base fluids as following [33,34]:

(1) $$k_{eff}=k_{Static}+k_{Brownian}$$

(2) $$k_{static}=k_{f}\left[\frac{\left(k_{s}+2k_{f}\right)\;-\;2\varphi \left(k_{f}-k_{s}\right)}{\left(k_{s}+2k_{f}\right)\;+\;\varphi \left(k_{f}-k_{s}\right)}\right]$$

(3) $$k_{brownian}\;=\;\left(5\times 10^{4}\right)\;\times \;\beta \varphi \rho_{f}Cp_{f}\sqrt{\frac{KT}{\rho_{s}d_{s}}}f\left(T,\varphi \right)$$

(4) $$\frac{\mu_{eff}}{\mu_{f}}=\frac{1}{1-34.87{\left(d_{s}/d_{f}\right)}^{-0.3}\varphi^{1.03}}$$

(5) $$d_{f}={\left[\frac{6M}{N\pi \rho_{fo}}\right]}^{1/3}$$

In the above, (*k_f_*), (*k_s_*), (*φ*), (β) refer to the base fluid and solid nanoparticles’ thermal conductivity, volume fraction, and thermal expansion. Meanwhile, (*K*) is the Boltzmann constant, (*T*) the working fluid temperature. Similarly, $\left(\mu_{f}\right)$ refer to the dynamic viscosity of the base fluid. (*d_s_/d_f_*) refers to the ratio between solid nanoparticle diameter to base fluid molecules diameter. (*M*) refers to the molecular weight of the working liquid, (*N*) is the Avogadro number, and $\left(\rho_{fo}\right)$ is the density of DW.

Meanwhile, the effective density and effective specific heat capacity are calculated from Equations (6) and (7) [34,35]:

(6) $$\rho_{eff}\;=\;\left(1-\varphi \right)\rho_{f}+\varphi \rho_{s}$$

(7) $${\left(Cp\right)}_{eff}=\frac{\left(1-\varphi \right){\left(\rho Cp\right)}_{f}+\varphi {\left(\rho Cp\right)}_{s}}{\left(1-\varphi \right)\rho_{f}+\varphi \rho_{s}}$$

Here, $\left(\rho_{eff}\right),\;(\rho_{s})$ and $\left(\rho_{f}\right)$ refer to the effective nanofluid density, solid nanoparticles density, and base fluid density, respectively. Similarly, $\left(Cp_{eff}\right),\;\left(Cp_{s}\right)$ and $\left(Cp_{f}\right)$ refer to the specific heat capacity of effective nanofluid, solid nanoparticles, and base fluid, respectively. Table 1 shows the thermophysical properties of the DW and metallic oxide nanoparticles.

### 2.2. Nanoparticles with Different Shapes

The above equations and formulas were designed for nanospherical particles and the effective thermal conductivity and effective dynamic viscosity of nanoplatelets, nano-blades, nanocylinders, and nanobricks will be estimated as per Equations (8)–(9) [37]. Meanwhile, the different shapes of nanoparticles did show impacts on the values of effective density and effective specific heat capacity of nanospherical nanofluids:

(8) $$\frac{k_{eff}}{k_{bf}}=1+\left(C_{K}^{Shape},+,C_{K}^{Surface}\right)\varphi \;=\;\left(1+C_{K}^{\varphi }\right)$$

(9) $$\mu_{eff}=\mu_{bf\times \left(1+A_{1}\varphi +A_{2}\varphi^{2}\right)}$$

Table 2 shows the different coefficients in Equations (8) and (9) for the effective thermal conductivity and effective dynamic viscosity.

## 3. Computational Method

### 3.1. Physical Model

**Figure 1:**
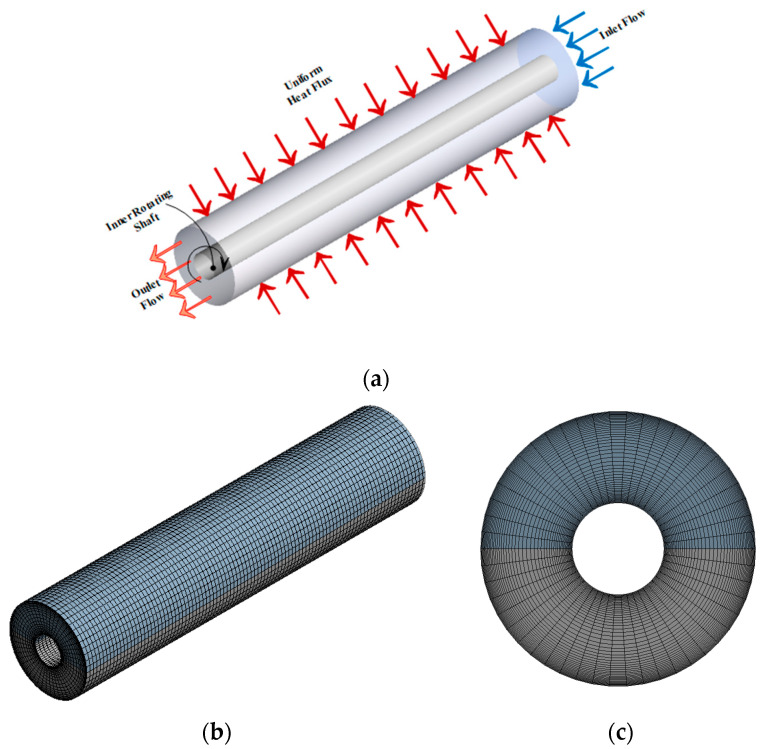
(**a**) Geometric model for the annular heat exchanger; (**b**,**c**) Computational meshing domain for 3D and front view of the model.

The computational model for the annuli pipes with the boundary conditions is illustrated in Figure 1a. The inner diameter of the annuli was (*D_in_* = 20 mm) with a wall thickness of (*t* = 5 mm), i.e., the hydraulic diameter of the annuli was (*D_h_ =* 10 mm). The total heated length of the annulus (*L*) was 400 mm. DW as a base fluid and various metallic oxide nanoparticles across multiple nanoparticle shapes were chosen as working fluids. Also, the thermal-physical properties of different nanofluids were temperature independent. The working fluids were in different velocities based on varied Reynolds number (Re) at the inlet boundary condition and exposed to pressure condition at the annuli outlet. The external cylinder pipe was subjected to constant heat wall flux. Meanwhile, the inner tube was kept insulated at a constant (*T_i_*).

The operating conditions of the annulus were based on several assumptions:

(i) The 3D annuli pipes operate under the condition of steady-state.
(ii) The heat transfer fluids are Newtonian and incompressible.
(iii) Working fluids flow under the conditions of single-phase and fully-developed.
(iv) The heat transfer losses are ignored.
(v) The thermal-physical properties of working fluids are evaluated at a constant temperature.

### 3.2. Governing Equations and Mathematical Model

As stated by many researchers [38,39], the finite volume method (FVM) can be used to solve partial differential equations (PDEs) by converting them to algebraic equations. The base fluid flows in similar velocity solid nanoparticles since the nanosuspensions were under the condition of a homogeneous single-phase flow, and therefore, and the effective thermal-physical properties of working fluids (DW and nanofluids) can be utilized to solve the transport equations such as continuity, momentum, and energy as below [40]:

*Continuity Equation:*

(10) $$\frac{1}{r}\frac{\partial }{\partial r}\left(\rho_{eff}rv_{r}\right)\;+\;\frac{1}{r}\frac{\partial }{\partial \theta }\left(\rho_{eff}rv_{\theta }\right)\;+\;\frac{1}{r}\frac{\partial }{\partial z}\left(\rho_{eff}rv_{z}\right)$$

*r−Momentum Equation:*

(11) $$\rho_{eff}\left(v_{r}\frac{\partial v_{r}}{\partial r}+\frac{v_{\theta }}{r}\frac{\partial v_{r}}{\partial \theta }-\frac{v\theta^{2}}{r}+v_{z}\frac{\partial v_{r}}{\partial z}\right)=\rho_{eff}g_{r}-\frac{\partial p}{\partial r}+u\left[\frac{\partial }{\partial r}\left(\frac{1}{r}\frac{\partial }{\partial r}\left(rv_{r}\right)\right)\right]+\frac{1}{r^{2}}\frac{\partial^{2}v_{r}}{\partial \theta^{2}}-\frac{2}{r^{2}}\frac{\partial v_{\theta }}{\partial \theta }+\frac{\partial^{2}v_{r}}{\partial z^{2}}$$

*θ−Momentum equation:*

(12) $$\rho_{eff}\left(v_{r}\frac{\partial v_{\theta }}{\partial r}+\frac{v_{\theta }}{r}\frac{\partial v_{\theta }}{\partial \theta }-\frac{v_{r}v_{\theta }}{r}+v_{z}\frac{\partial v_{\theta }}{\partial z}\right)=\rho_{eff}g_{\theta }-\frac{1}{r}\frac{\partial p}{\partial \theta }+u\left[\frac{\partial }{\partial r}\left(\frac{1}{r}\frac{\partial }{\partial r}\left(rv_{\theta }\right)\right)\right]+\frac{1}{r^{2}}\frac{\partial^{2}v_{\theta }}{\partial \theta^{2}}-\frac{2}{r^{2}}\frac{\partial v_{r}}{\partial \theta }+\frac{\partial^{2}v_{\theta }}{\partial z^{2}}$$

*z−Momentum equation:*

(13) $$\rho_{eff}\left(v_{r}\frac{\partial v_{z}}{\partial r}+\frac{v_{\theta }}{r}\frac{\partial v_{z}}{\partial \theta }+v_{z}\frac{\partial v_{z}}{\partial z}\right)=\rho_{eff}g_{z}-\frac{\partial p}{\partial z}+u\left[\left(\frac{1}{r}\frac{\partial }{\partial r}\left(r\frac{\partial v_{z}}{\partial r}\right)\right)\right]+\frac{1}{r^{2}}\frac{\partial^{2}v_{z}}{\partial \theta^{2}}+\frac{\partial^{2}v_{z}}{\partial z^{2}}$$

*Energy Equation:*

(14) $$\rho_{eff}cp_{eff}\left(v_{r}\frac{\partial T}{\partial r}+\frac{v_{\theta }}{r}\frac{\partial T}{\partial \theta }+v_{z}\frac{\partial T}{\partial z}\right)=k_{eff}\left[\frac{1}{r}\frac{\partial }{\partial r}\left(r\frac{\partial T}{\partial r}\right)+\frac{1}{r^{2}}\frac{\partial^{2}}{\partial \theta^{2}}+\frac{\partial^{2}T}{\partial z^{2}}\right]\;+\;\mu_{eff}\varphi$$

The current problem is considered as a fully-developed flow, and the (*k-ε*) turbulent model was applied [38,41,42]. As per Equations (15)–(16), the turbulent kinetic energy and specific rate of dissipation are written as follows:

(15) $$\nabla .\left(\rho kV\right)\;=\;\nabla .\left[\left(\frac{\mu_{t}}{\sigma_{k}}\right)\nabla \left(k\right)\right]\;+\;G_{k}-\rho \varepsilon$$

(16) $$\nabla .\left(\rho \varepsilon V\right)\;=\;\nabla .\left[\left(\frac{\mu_{t}}{\sigma_{\varepsilon }}\right)\nabla \varepsilon \right]+\frac{\varepsilon }{k}\left(\;C_{1\varepsilon }G_{k}+\varepsilon \;C_{2\varepsilon }\rho \right)$$

where, $\left(G_{k}\right)$ means the generation of turbulent kinetic energy as a result of mean velocity gradient and $\mu_{t}=\rho C_{\mu }^{k^{2}}/\varepsilon$ is the term of turbulence viscosity. (ε) refers to a specific Rate of dissipation for kinetic energy. The values of the constants in Equation (16) $C_{\mu }$, $C_{1\varepsilon }$, $C_{2\varepsilon }$, $\sigma_{k}$, and $\sigma_{\varepsilon }$ are 0.09, 1.44, 1.92, 1.0, and 1.3, respectively.

Moreover, the dimensionless quantities used in this study (Nu_avg_, f and Re) are written in Equations (17)–(20). The simulation data using pure water were validated with the equations of Dittos-Boelter (Equation (17)) for *Nu_avg_* and Blasius (Equation (19)) for friction factor (*f*) [38]. For all the governing equations, the convergence solutions were set for residuals <10^−6^.

(17) $$Nu_{avg}=\frac{h_{f}}{k_{f}}D_{eff}=0.023Re^{0.8}Pr^{0.4}$$

(18) $$Nu_{avg}=\frac{hD_{h}}{k_{eff}}$$

(19) $$f=\frac{0.316}{Re^{0.25}}$$

(20) $$Re=\frac{UD_{h}\rho_{eff}}{\mu_{eff}}$$

In this regard, $\left(U\right)$, $\left(D_{h}\right)$, $\left(\rho_{eff}\right)$ and $\left(\mu_{eff}\right)$ are the working fluid velocity, hydraulic diameter, effective density, and effective dynamic viscosity, respectively. Moreover, $\left(k_{eff}\right)$ refers to effective thermal conductivity.

### 3.3. Boundary Conditions

The thermal-physical properties of different metallic oxides nanofluids in different shapes were evaluated under the conditions of varying volume concentrations (1%-vol., 2%vol., 3%-vol., and 4%-vol.) at 293 K. Numerical studies were performed with a steady velocity profile at the inlet boundary condition and pressure-outlet condition used at the outlet of the annuli tube. Turbulent intensity formula (I = 0.16 × Re ^−(1/8)^) was stated for the first value of both turbulent quantities (*k* and *ε*).

The boundary conditions of the problem are given by [43]:

(21) $$z\;\geq \;0\;and\;r=R_{i},\;v=u=0\;and\;w=R_{i}\omega \;z\;\geq \;0\;and\;r=R_{o},\;v=u=w=0\;z=0\;and\;R_{i}<r<R_{o},\;u=u_{o}\;\mathrm{At}\;z=0,\;p=p_{o}$$

### 3.4. Grid Independence Test and Code Validation

**Figure 2:**
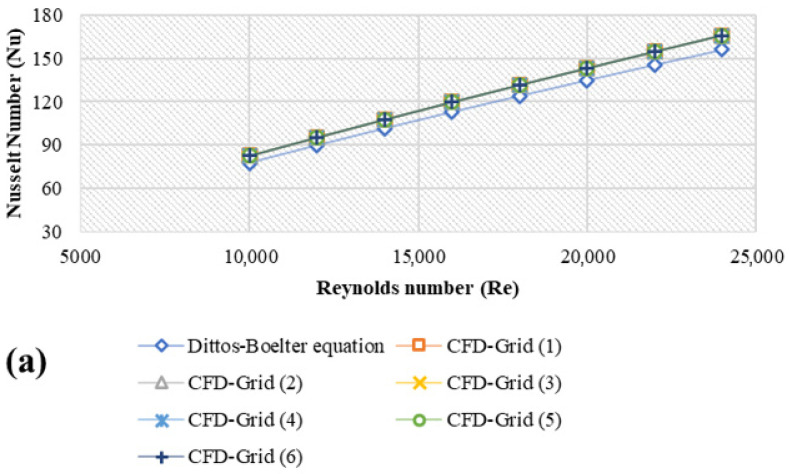

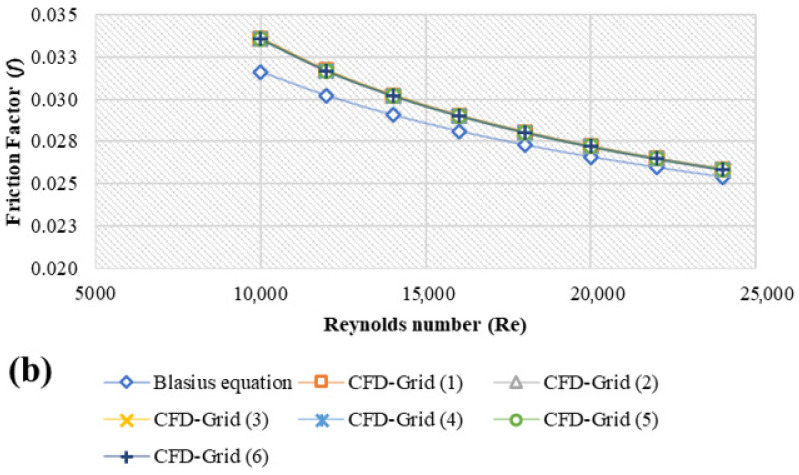
Grid Independence Test against different Reynolds numbers and different CFD grid sizes at 293 K; (**a**) Average Nusselt number, (**b**) friction factor.

The SIMPLE algorithm was applied to solve the pressure-velocity coupling, while the second-order-upwind scheme was using for solving the equations of pressure, momentum, turbulent kinetic energy, turbulent dissipation rate, and energy. The structured uniform grid distribution was utilized to discretize the computation zones (see Figure 1b,c). Although the velocity and temperature gradients are high, it is finer near the annulus entry and the wall. Six distinct grid distributions were put to the test to confirm that the estimated findings were accurate. Increases in the grid sizes in the θ-direction, r-direction, and z-direction have no significant effect on Nu_avg_ and f at the outer wall boundary condition, as shown in Figure 2. As a result, for the current calculations in the axial (*z*), tangential (*θ*), and radial (*r*) directions, the grid of 350 × 30 × 30 elements were chosen.

**Figure 3:**
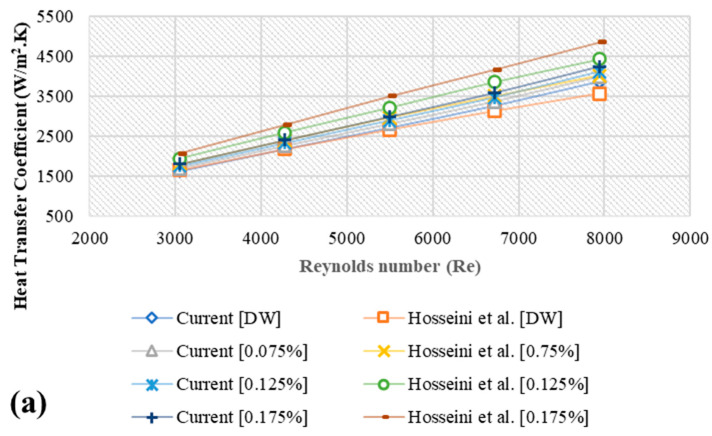

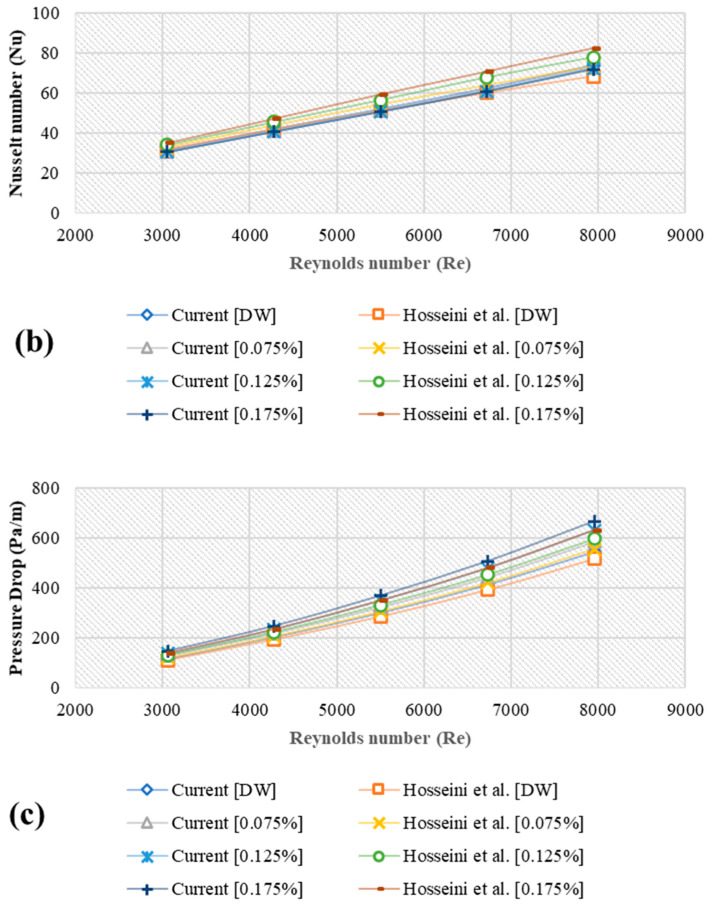
Comparison of the results of the current study with those of Hosseini et al. [12]; (**a**) Heat transfer coefficient, (**b**) Nusselt number, (**c**) Pressure loss.

The numerical validation method entails simulating numerical codes for benchmark issues under certain conditions. These simulation results are compared to experimental data published in the literature. This should produce results that are identical to or extremely similar to those obtained in earlier studies. The study of Hosseini et al. [12] was used to validate the current findings. Their report discussed the effects of using MWCNTs suspended in DW on the values of heat transfer and thermal-physical properties in an annular heat exchanger. As per Figure 3, it was noted that, the average error was 6.2% (Figure 3a,b and 5.5% as in Figure 3c).

## 4. Results and Discussion

### 4.1. Morphologies of Spherical Nanoparticles

**Figure 4:**
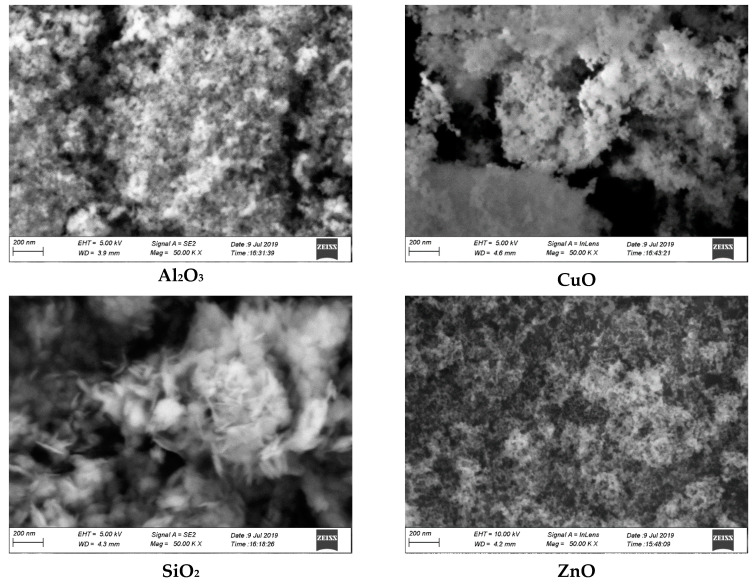
FE-SEM images of different metal oxides nanostructures at the nanoscale of 200 nm.

For this study, four different dry nanoparticles were purchased from Sigma-Aldrich (Selangor, Malaysia) (M) SDN BHD namely; Al_2_O_3_, CuO, SiO_2_ and ZnO. First, the produced nanosuspensions were characterized by using a field-emission scanning electron microscope (FE-SEM, (Sonics Vibra-Cell, VC 750, Sonics & Materials, Inc., Newtown, CT, USA)) to identify the equal dispersion of all metal oxide nanoparticles in the base fluid. Then, an ultrasonic probe (FE-SEM, ZEISS Sigma, Oberkochen, Germany) was applied for 1 hr. to obtain a homogenous distribution of nanoparticles and break down any large agglomerates [44]. Regular use of the ultrasonic probe or a magnetic stirrer decreases nanoparticle agglomeration. Nanoparticles tend to aggregate due to their high surface area [45,46]. Therefore, investigators recommended a two-step technique for preparing oxide nanofluids over those with metallic nanofluids [47,48,49]. The two-step technique is standard as the most cost-effective procedure for making nanofluids [50]. Figure 4 displays that the four samples have negligible agglomeration, and they presented better suspension. Also, FE-SEM images showed that the metallic oxide nanoparticles were found to be well dispersed and primarily spherical in shape. Meanwhile, the particles were in the nano-sizes of Al_2_O_3_, 13 nm, CuO, <50 nm, SiO_2_, 10–20 nm and ZnO, <100 nm.

### 4.2. Thermophysical Properties of Different Nanostructures

**Figure 5:**
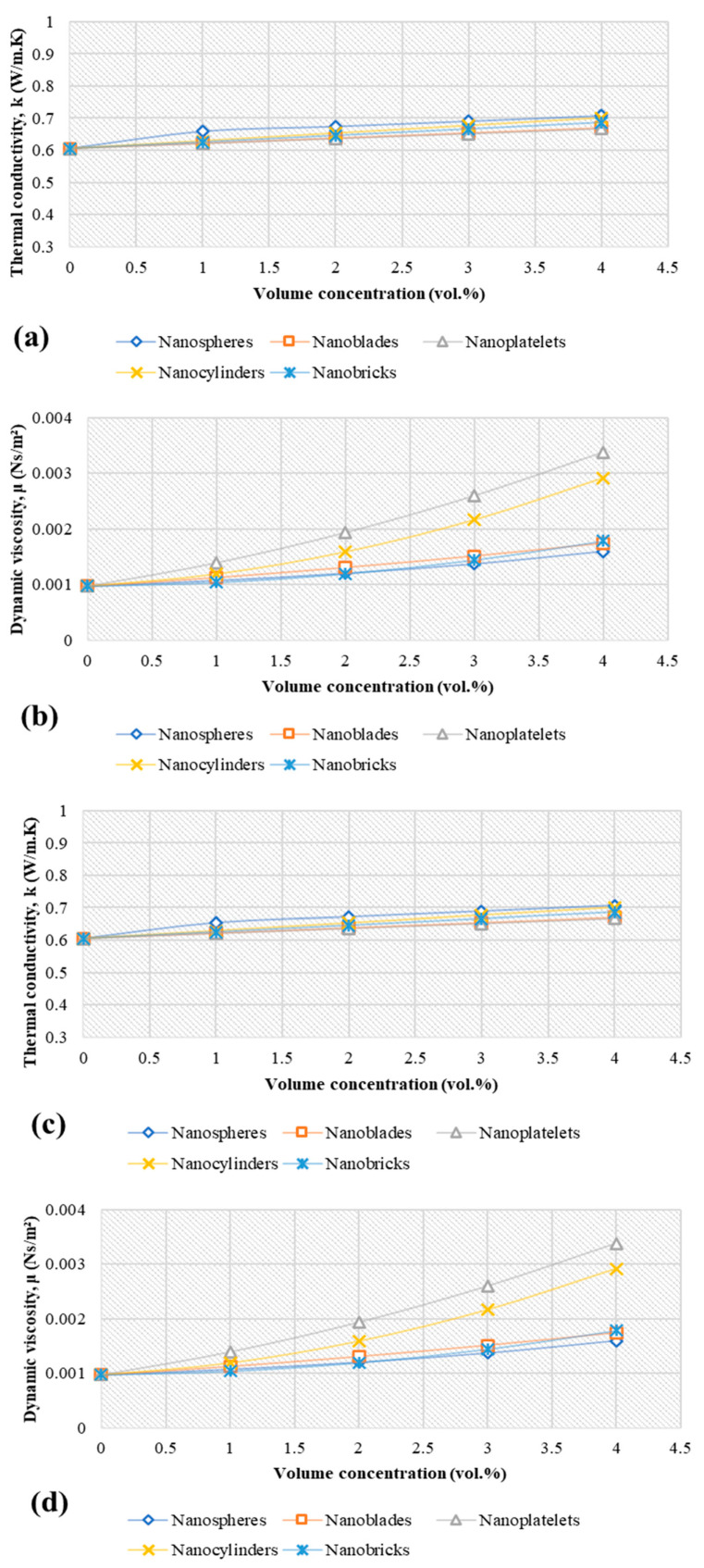

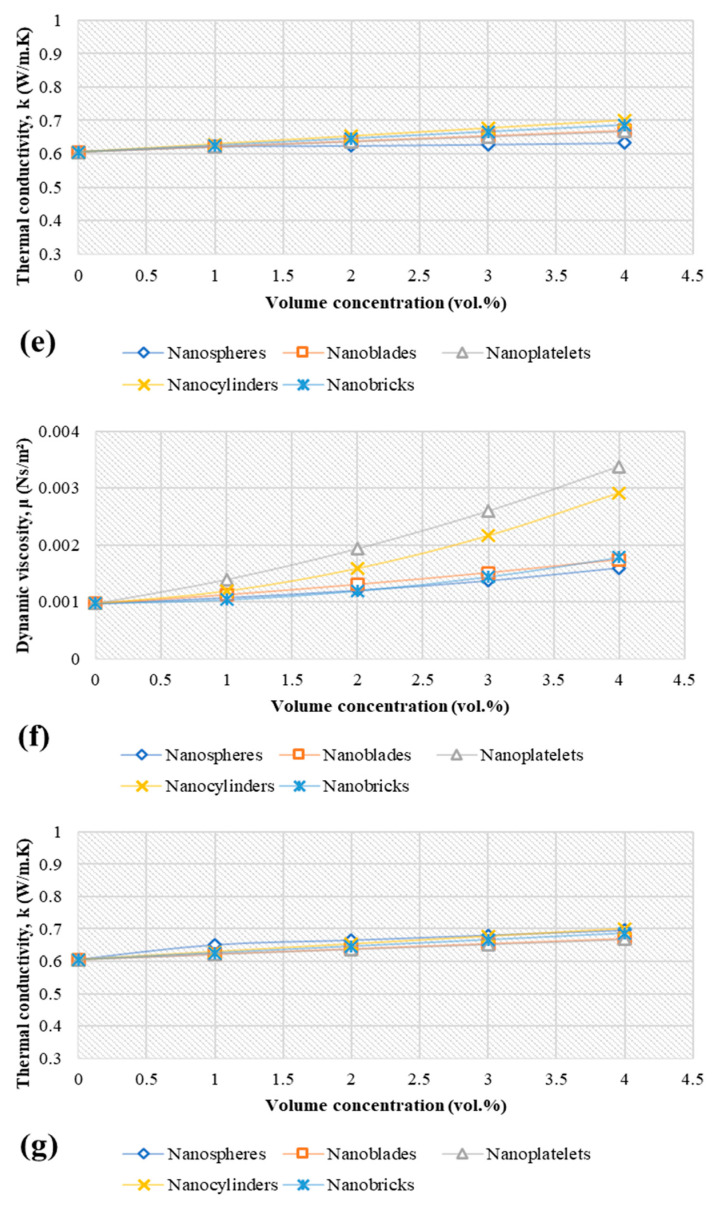

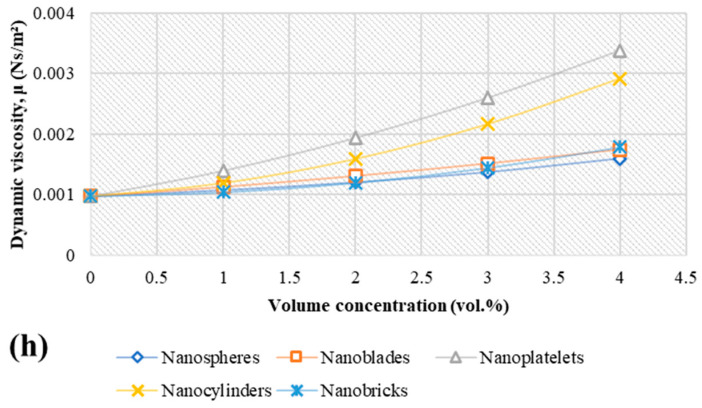
Thermal conductivity and dynamic viscosity for different metal oxide nanostructures and different nanoparticles shape at 20 nm and 293 K; (**a**) k-Al_2_O_3_, (**b**) µ-Al_2_O_3_, (**c**) k-CuO, (**d**) µ-CuO, (**e**) k-SiO_2_, (**f**) µ-SiO_2_, (**g**) k-ZnO, (**h**) µ-ZnO.

Figure 5 presents the estimated effective thermal conductivity and effective dynamic viscosity of the different nanofluids at nanoparticle size of 20 nm and temperature of 293 K. Figure 5a,b show the nanofluids’ thermal conductivity and dynamic viscosity profiles versus the various volume fractions of alumina nanofluid, respectively. The nanospherical shape showed the highest thermal conductivity due to the uniform distribution of Van der Waals forces along their surface, leading to a better suspension and better contact between the nanoparticles and pure water. In addition, the influence of particle-host fluid homogeneity was visible in viscosity, with spherical shape nanoparticles showing the lowest percentage increase in viscosity compared to the base fluid of all nanoparticle shapes.

The thermal conductivity of spheres-Al_2_O_3_ and spheres-CuO was enhanced by (8.98%, 11.53%, 14.28%, 17.14%) and (8.05%, 10.96%, 13.85%, 16.74%) for 1 vol.%, 2 vol.%, 3 vol.% and 4 vol.%, respectively. Also, the thermal conductivity of SiO_2_ spheres and ZnO spheres was enhanced by (2.72%, 3.13%, 3.83%, 4.62%) and (7.53%, 9.91%, 12.45%, 15.08%) for 1 vol.%, 2 vol.%, 3 vol.% and 4 vol.%, respectively. Meanwhile, the increments in thermal conductivity of nanoblades, nanoplatelets, nanocylinders and nanobricks for all nanoparticles type were (2.74%, 5.48%, 8.22%, 10.96%), (2.61%, 5.22%, 7.83%, 10.44%), (3.95%, 7.90%, 11.85%, 15.80%) and (3.37%, 6.74%, 10.11%, 13.48%), respectively, relative to DW. As per equations (4) and (9), the increase in dynamic viscosity was unaffected by the type of nanofluid used, but depended on the shape of the nanoparticles. In this regard, the increments were (10.24%, 23.41%, 40.45% and 63.21%), (15.83%, 34.13%, 54.90%, 78.13%), (43.23%, 98.70%, 166.43%, 246.42%), (22.54%, 63.18%, 121.90%, 198.70%) and (6.61%, 22.66%, 48.13%, 83.02%) for spheres, blades, platelets, cylinders and bricks, respectively, for all types of materials, at various concentrations.

### 4.3. Heat Transfer and Hydrodynamic Properties

**Figure 6:**
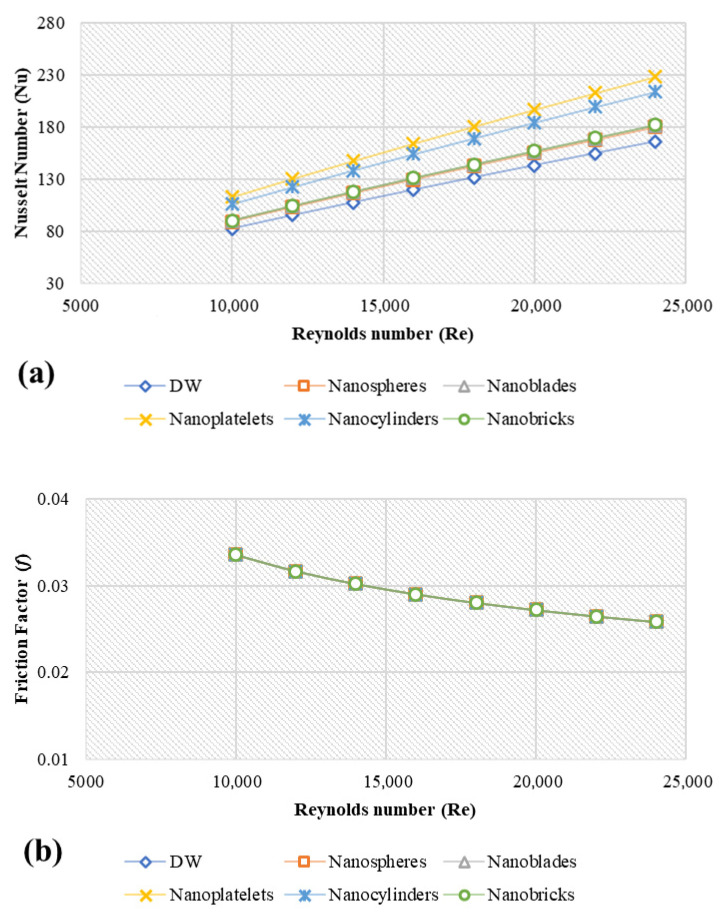

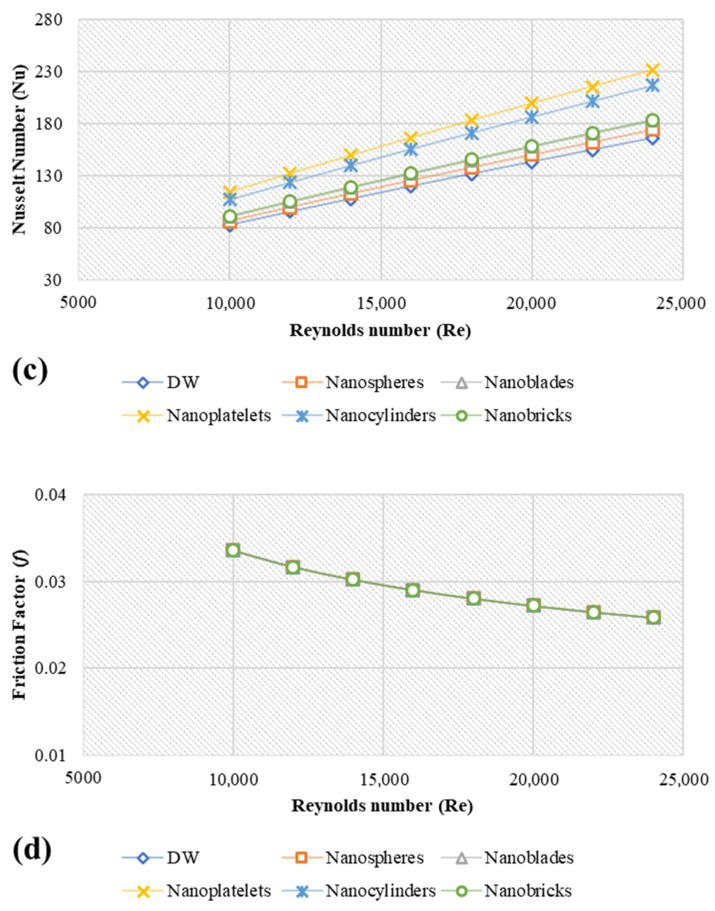

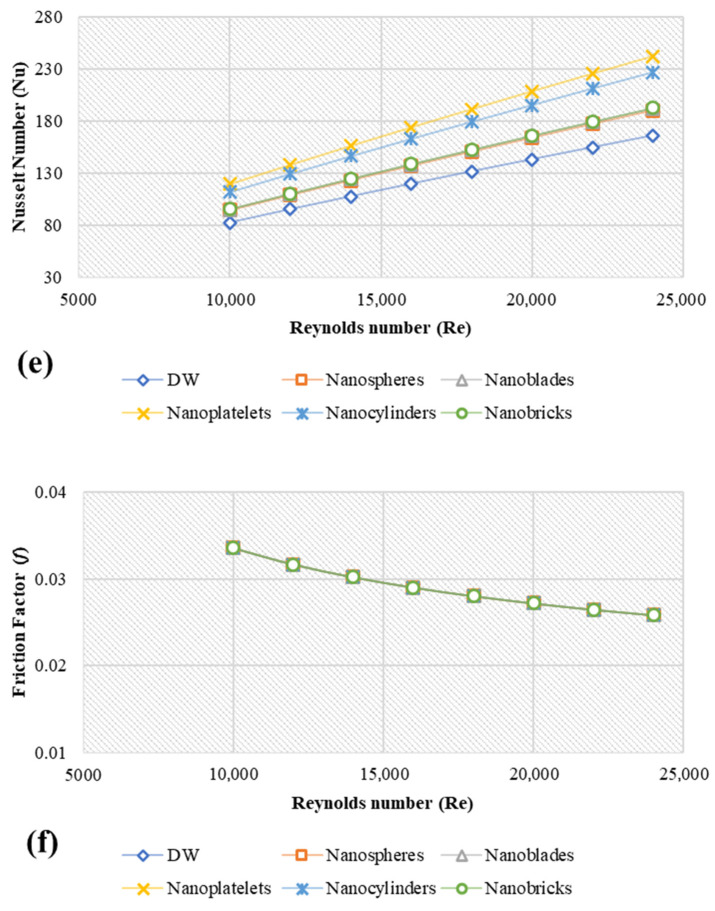

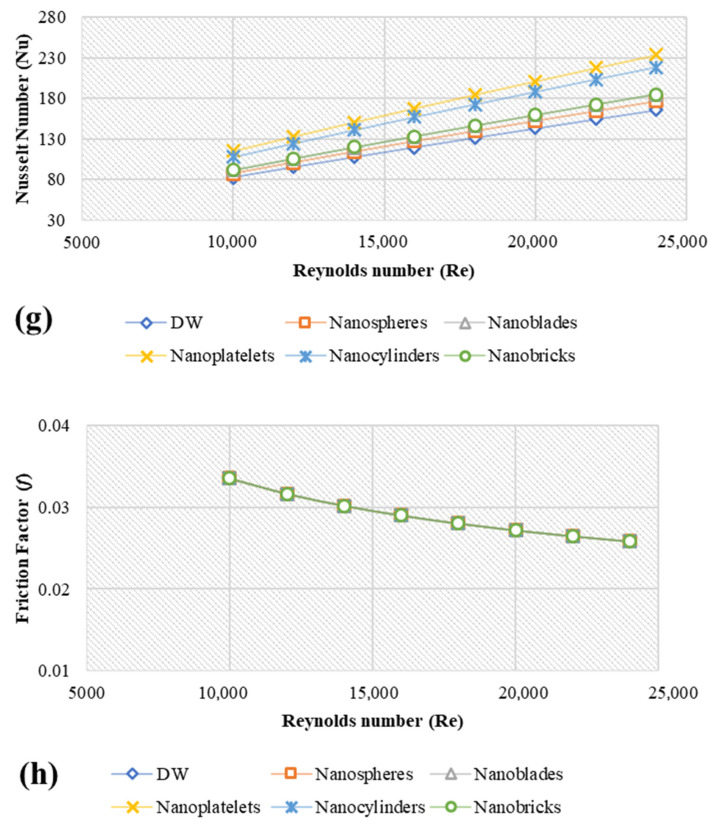
Simulation data of average Nusselt numbers and friction factors for different metal oxides and different nanoparticles shape at 4 vol.%, 20 nm, and 293 K; (**a**) Nu-Al_2_O_3_, (**b**) *f*-Al_2_O_3_, (**c**) Nu-CuO, (**d**) *f*-CuO, (**e**) Nu-SiO_2_, (**f**) *f*-SiO_2_, (**g**) Nu-ZnO, (**h**) *f*-ZnO.

Figure 6 shows the average Nusselt number (Nu_avg_) and friction factor (*f*) of different metal-oxide nanostructures under the conditions of 4 vol.%, 20 nm, 293 K and 5000 W/m^2^. In all scenarios, the Nusselt number rises as the Reynolds number rises. Inflow velocity increases due to the increase in the Re since the cross-sectional area and hydraulic diameter are constant. As the inflow velocity rises, the fluid’s residence time lowers, resulting in a lower output temperature. On the other hand, increased input velocity increases the convective heat transfer coefficient, resulting in larger Nusselt number values and increased heat transfer [51]. Figure 6a shows the Nu number profiles of Alumina at different Re number where, the average enhancements were 8.47%, 9.51%, 37.69%, 28.91% and 9.69% for the nanosphere, nanoblade, nanoplatelet, nanocylinder and nanobrick shapes, respectively. The enhancement percentages of Nu number can be attributed to the effect of the Prandtl number ($Pr=\frac{\mu_{eff}\times Cp_{eff}}{k_{eff}}$) of each nanofluid sample where the highest Pr the highest Nu_avg_ at the same Re, and these results match with the published work of Abdelrazek et al. [27]. Figure 6b shows the variation of (*f*) versus Re for the different alumina nanostructures compared to the DW. As shown in Figure 6b, the results proved that the friction factor of nanofluids is Reynolds number-dependent, as stated in Equation (19), indicating that nanofluids can be considered single-phase fluids. Other nanofluids of CuO nanostructures confirmed the same findings of the friction factor for alumina nanostructures in Figure 6d, silica nanostructures in Figure 6f, and ZnO nanostructures in Figure 6h.

CuO nanofluids show about 4.96%, 10.41%, 39.45%, 30.46% and 10.51% for nanospheres, nanoblades, nanoplatelets, nanocylinders, and nanobricks, respectively. Meanwhile, the average enhancement in Nu using SiO_2_ was 14.66%, 15.84%, 45.91%, 36.57%, 16.03% for nanospheres, nanoblades, nanoplatelets, nanocylinders and nanobricks, respectively. Also, the Nusselt number enhancement for ZnO nanofluids was 6.42%, 11.33%, 40.54%, 31.50% and 11.52% using nanospheres, nanoblades, nanoplatelets, nanocylinders and nanobricks, respectively. Figure 6 indicates that, SiO_2_ nanofluids show the higher heat transfer enhancement followed by ZnO, CuO, and Al_2_O_3_. While platelets, nanoparticles show the highest reading, followed by cylinders, bricks, blades, and spheres. For friction factor, only Al_2_O_3_ nanofluids show slight variations by about 6.93% with DW data using nanoblade, nanoplatelet, nanocylinder and nanobrick shapes. Meanwhile, the condition was not applicable for nanospheres-Al_2_O_3_. CuO, SiO_2,_ and ZnO in different nanoparticle shapes did not present any significant differences with the water data. Temperature and velocity contours of different nanofluids and different nanoparticles shape under the conditions of 4 vol.%, 20 nm, 293 K, Re = 10,000 and 5000 W/m^2^ are presented in Figure S1 in Supplementary Materials.

### 4.4. Effect of Heat Flux Ratio

**Figure 7:**
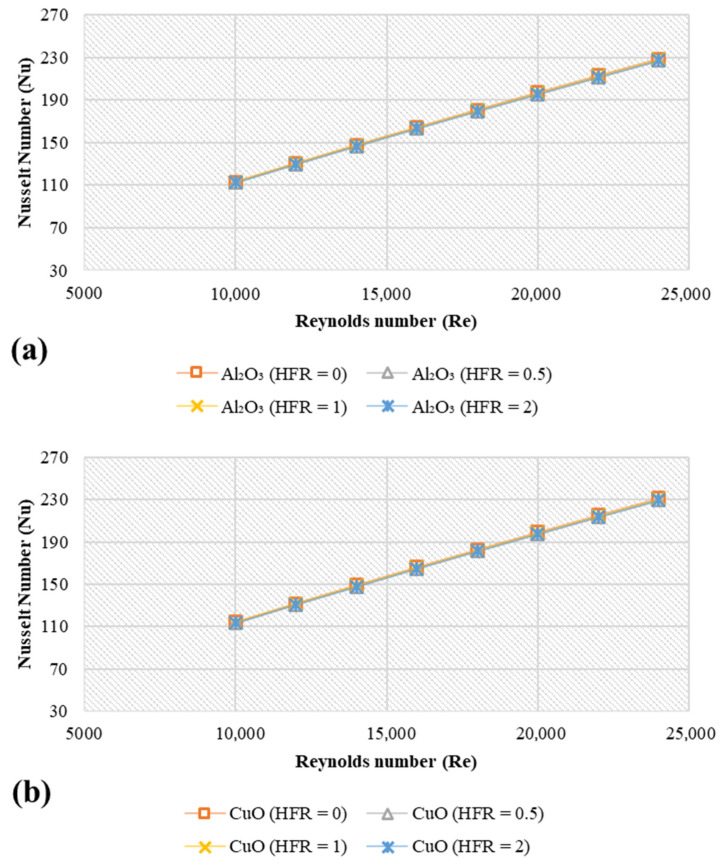

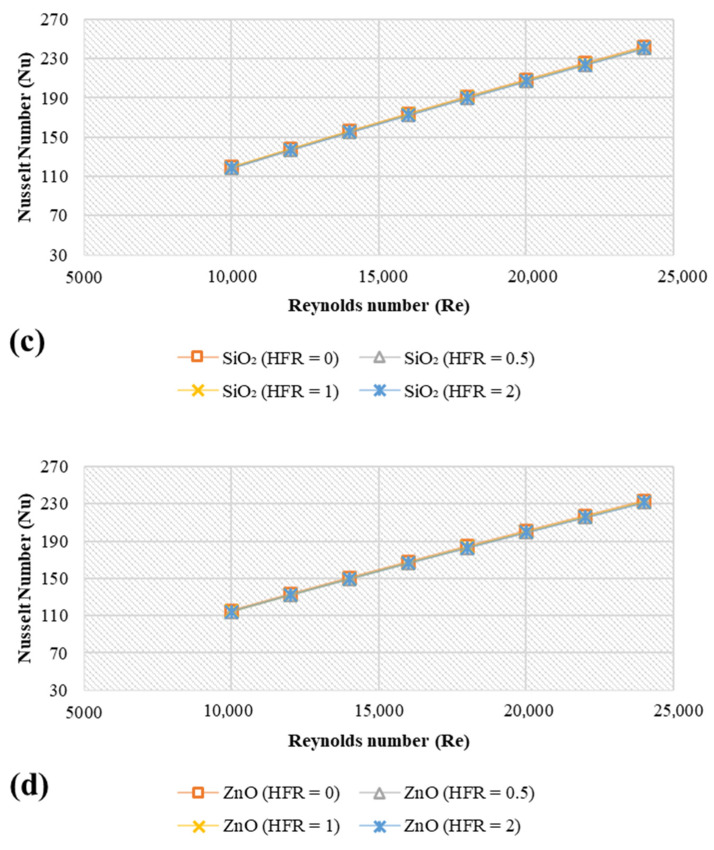
Simulation data of average Nusselt number for different metal oxides and different heat flux ratios at 4 vol.%, 20 nm, and 293 K; (**a**) Nanoplatelets -Al_2_O_3_, (**b**) Nanoplatelets -CuO, (**c**) Nanoplatelets -SiO_2_, (**d**) Nanoplatelets -ZnO.

Figure 7 illustrates the Nusselt number of different heat flux ratios (HFR) and different nanofluids at the conditions of 4 vol.%, 20 nm, 293 K, and platelet nanoparticles. The values of heat flux ratio ($q_{i}/q_{o}$) ranged from 0 to 2. As can be seen in Figure 7, the effect of HFR did not show significant impacts on the values of Nu of different nanofluids, which matches the basic knowledge of convection heat transfer. The heat transfer rates can be changed when the working fluid bulk temperature changes. The working fluid bulk temperature results from different values of heat source at the inner and outer pipes. In the current case, the bulk temperature is very close to the inner wall temperature. Moreover, the contours of temperature for different heat flux ratios (HFR) and different nanoparticle shapes at the conditions 4 vol.%, 20 nm, 293 K and Re = 10,000 are reported in Figure S2 in Supplementary Materials.

### 4.5. Effect of Inner Shaft Rotation (ω)

**Figure 8:**
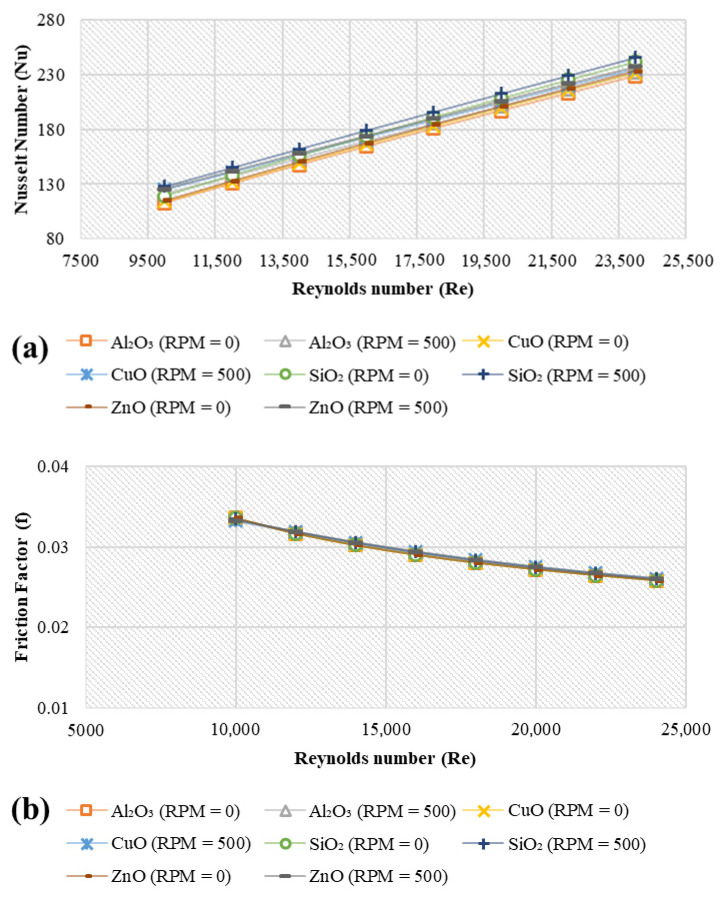
Variations of (**a**) average Nusselt number and (**b**) friction factor for different metal oxide nanostructures and different rotation speeds at 4 vol.%, 20 nm, and 293 K.

Figure 8 presents the values of the average Nusselt number and friction factor of different nanofluids with and without the inner cylinder rotation (500 RPM and 0 RPM). With an inner shaft rotation speed of 500 RPM, the average heat transfer was enhanced by (37.28% to 42.62%), (38.90% to 45.58%), (45.27% to 50.44) and (40.13% to 45.04%) for Al_2_O_3_, CuO, SiO_2_ and ZnO, respectively, under the conditions of 4 vol.%, 20 nm, 293 K, and platelet nanoparticles. The enhancements can be credited to Taylor vortices [52], which are developed after the entry region. The vortices interrupt the steady development of the boundary layers and improve the annular boundary’s heat transfer coefficients in forced convection. As a result, the turbulent kinetic energy improved as the rotation speed increased [53], resulting in a significant heat transfer and momentum improvement. Meanwhile, only Al_2_O_3_ nanofluids showed substantial differences in friction factor values for the two cases of 0 RPM and 500 RPM by about 6.72% and 6.15% relative to DW.

### 4.6. Effect of Concentric Aspect Ratio

**Figure 9:**
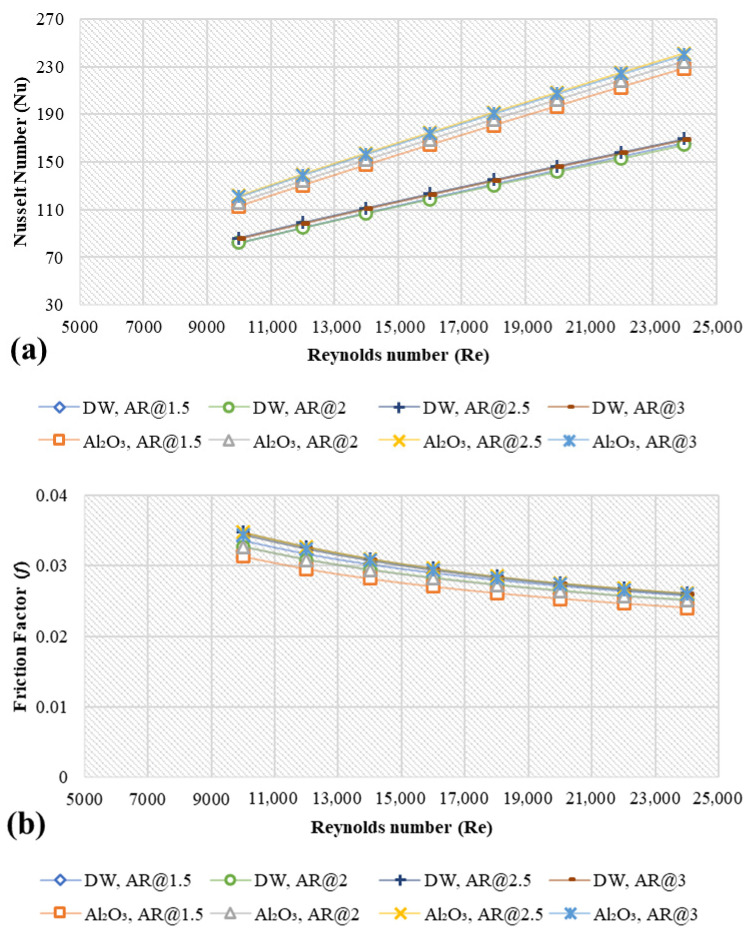

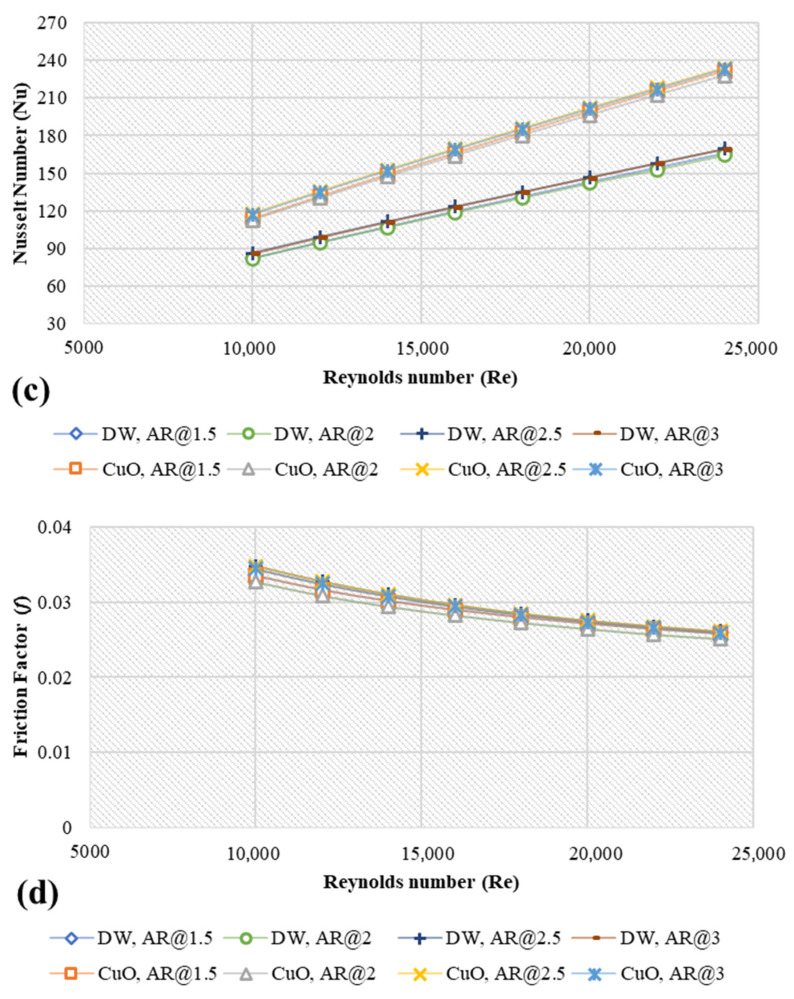

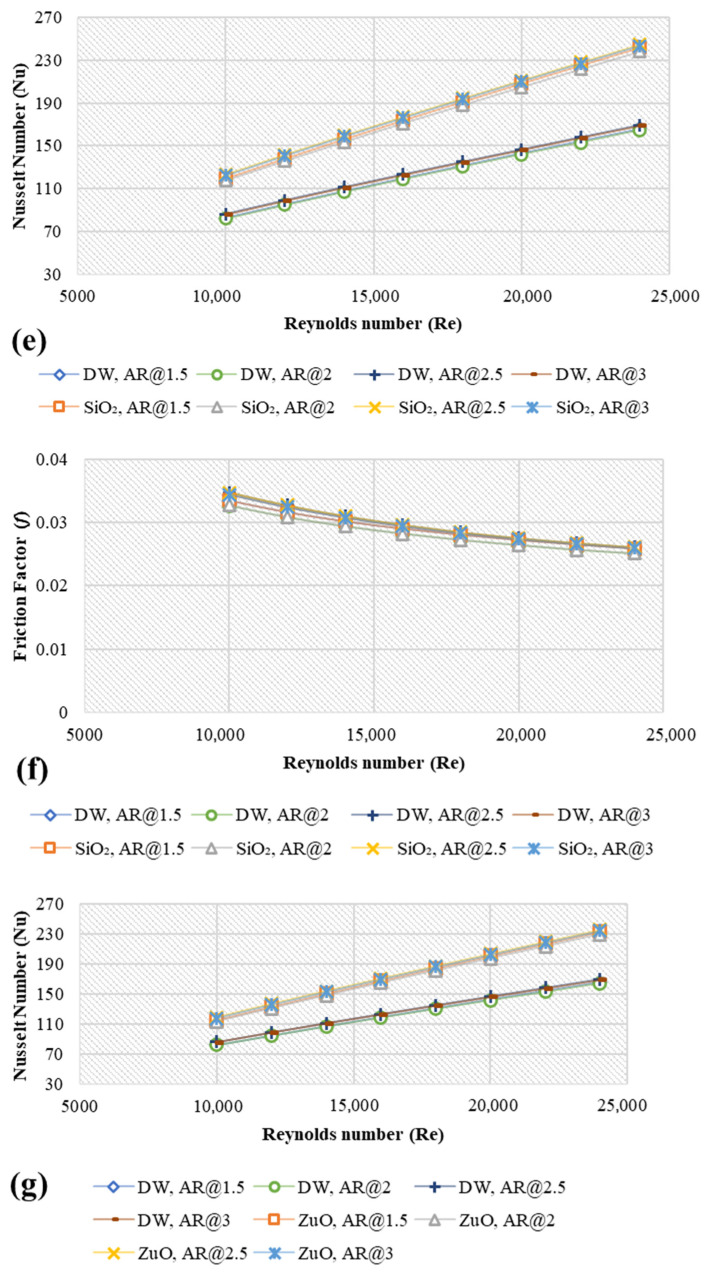

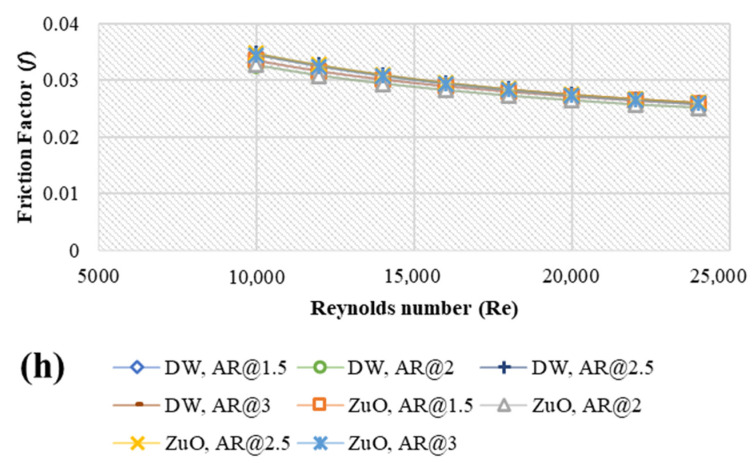
Simulation data of average Nusselt number and friction factor for different metal oxides and different aspect ratios at 4 vol.%, 20 nm, and 293 K; (**a**) Nu-Al_2_O_3_, (**b**) *f*-Al_2_O_3_, (**c**) Nu-CuO, (**d**) *f*-CuO, (**e**) Nu-SiO_2_, (**f**) *f*-SiO_2_, (**g**) Nu-ZnO, (**h**) *f*-ZnO.

The annulus aspect ratio (*D_o_/D_i_*) effect is also studied and depicted in Figure 9. The radial component of the annulus is not simulated since the hydraulic diameter is only considered in the axial direction. Relative to pure water, as the annulus aspect ratio (*AR*) increased from 1.5, 2, 2.5 and 3, the average enhancements in the heat transfer were (37.28%, 42.12%, 41.67%, 41.39%), (38.97%, 37.94%, 37.51%, 37.24%), (45.35%, 44.11%, 43.68%, 43.38%) and (40.05%, 38.95%, 38.56%, 38.29%) for Al_2_O_3_, CuO, SiO_2_ and ZnO, respectively. Figure 9 clarifies the influence of dispersed metal oxides nanoparticles on the heat transfer properties of the base fluid owing to Brownian motion, which increases energy transfer within the fluid (diffusion) and then increases the rate of heat transfer from the wall to the next stagnant layer of the fluid (conduction). The heat transfer rate increases in the thermal applications due the increments in the diffusivity and Prandtl number [27]. In this regard, AR = 2 and SiO_2_ nanofluids showed a higher value of heat transfer enhancements. Moreover, SiO_2_ has the lowest thermal conductivity than other nanofluids but higher than base fluid and has the highest average velocity among other working fluids due to its lowest density. The heat transfer improves by using higher hydraulic diameter ratios, and the problems due to heating and cooling in the industry can be reduced. High hydraulic diameter ratio gave a small hydraulic diameter, then the risks of sedimentations of nanofluids and abrasion on annulus can be reduced. Meanwhile, the different aspect ratios did not show significant impacts on the values of friction factors. Figure S3 in Supplementary Materials shows the DW temperature and velocity contours and different nanofluid types (Al_2_O_3_, CuO, SiO_2,_ and ZnO) at 4 vol.%, 20 nm, 293 K and Re = 10,000.

## 5. Conclusions

Heat transfer and hydrodynamic properties of a turbulent convection flow were numerically simulated using in three-dimensional horizontal concentric annuli. According to the current study, the following conclusions can be made:

(i) Six different grids were tested, but 350 × 30 × 30 elements were selected for the present calculations.
(ii) FE-SEM analysis showed that, Al_2_O_3_, CuO, SiO_2,_ and ZnO were well dispersed and found to be predominantly spherical.
(iii) At 4 vol.%, the best enhancements in thermal conductivity were 17.14% (spheres-Al_2_O_3_), 16.74% (spheres-CuO), 15.80% (bricks-SiO_2_) and 15.08% (spheres-ZnO). Meanwhile, ZnO presented a sharp increment in the viscosity for all nanoparticle shapes.
(iv) SiO_2_ nanofluids showed a higher heat transfer enhancement, followed by ZnO, CuO, and Al_2_O_3_. In comparison, platelet nanoparticles show the highest reading, followed by cylinders, bricks, blades, and spheres. Different metallic oxides and different nanoparticle shapes did not show significant variations of friction factor.
(v) The effect of HFR did not show significant impacts on the values of Nu of different nanofluids.
(vi) With an inner shaft rotation speed of 500 RPM, the average heat transfer enhanced by (37.28% to 42.62%), (38.90% to 45.58%), (45.27% to 50.44) and (40.13% to 45.04%) for Al_2_O_3_, CuO, SiO_2_ and ZnO, respectively, at the conditions of 4 vol.%, 20 nm, 293 K, and platelets nanoparticles. Meanwhile, only Al_2_O_3_ nanofluids showed any significant differences in friction factor values.
(vii) AR = 2 and nanoplatelets-SiO_2_ nanofluids showed the higher value of heat transfer enhancements of 43.68% at 4 vol.%, 20 nm, 293 K.

## Figures and Tables

**Table 1:** Values of thermophysical properties for DW and metallic oxides [33,35,36].

Nanoparticle/DW	ρ (kg/m^3^)	Cp (J/kg. K)	μ (N.s/m^2^)	k (W/m. K)
*Al_2_O_3_*	3970	765	-	40
*CuO*	6500	535.6	-	20
*SiO_2_*	2200	703	-	1.2
*ZnO*	5600	495.2	-	13
*DW*	997.78	4076.4	0.0009772	0.60475

**Table 2:** Coefficients of thermal conductivity and dynamic viscosity of different nanoparticle shapes [37].

Nanoparticle Shapes	AR	$C_{K}^{Shape}$	$C_{K}^{Surface}$	C_K_	A_1_	A_2_
Nanoplatelets	1:1/8	5.72	−3.11	2.61	37.1	612.6
Nanoblades	1:6:1/12	8.26	−5.52	2.74	14.6	123.3
Nanocylinders	1:8	4.82	−0.87	3.95	13.5	904.4
Nanobricks	1:1:1	3.72	−0.35	3.37	1.9	471.4

## Data Availability

The data presented in this study are available on request from the corresponding author.

## Supplementary Materials

The following are available online at www.mdpi.com/article/10.3390/nano11081979/s1, Figure S1: Temperature and velocity contours of different nanofluids and different nanoparticles shape under the conditions of 4 vol.%, 20 nm, 293 K, Re = 10,000 and 5000 W/m^2^, Figure S2: Temperature contours of different nanofluids and different heat flux ratios (HFR) under the conditions of 4 vol.%, 20 nm, 293 K, Re = 10,000 and 5000 W/m^2^, Figure S3: Temperature and velocity contours of DW and different nanofluid types (Al2O3, CuO, SiO2, and ZnO) at 4 vol.%, 20 nm, 293 K and Re = 10,000.

## Abbreviations

*Ag*	Silver	*k*	Thermal Conductivity, [W/m. K]
*Al_2_O_3_*	Aluminum Oxide	*_K_*	Boltzmann constant
*AR*	Aspect ratio, [D_o_/D_i_]	*L*	Total length of annuli, [mm]
*C_p_*	Specific Heat capacity, [KJ/kg. K]	*M*	Molecular Weight
*Cu*	Copper	*N*	Avogadro number
*CuO*	Copper Oxide	*NSE*	Navier Stokes Equations
*D_h_*	Hydraulic Diameter, [mm]	*Nu_avg_*	Average Nusselt Number
*D_i_*	Inner Pipe Diameter, [mm]	*Pr*	Prandtl Number
*D_o_*	Outer Pipe Diameter, [mm]	*q_w_*	Pipe Heat Flux, [W/m^2^]
*EG*	Ethylene Glycol	*R_e_*	Reynolds number
*FVM*	Finite Volume Method	*SiO_2_*	Silicon dioxide
*Gr*	Grashof number	*T_in_*	Inner Cylinder Temperature, [K]
*h*	Heat transfer coefficient, [W/m^2^. K]	*U_o_*	Velocity inlet, [m/s]
*HFR*	Heat Flux Ratio, [q_i_/q_o_]	*ZnO*	Zinc Oxide
**Greek alphabet symbols & letters**			
*α*	Thermal diffusivity, [m^2^/s]	*β*	Thermal expansion coefficient, [1/K]
*ε*	Turbulent dissipation rate, [m^2^/s^2^]	*μ*	Working fluid viscosity, [N. m/s]
*ν*	Kinematic viscosity, [m^2^/s]	*ρ*	Density, [kg/m^3^]
*φ*	Volume fraction [vol.%]		
**Indexes**			
*bf*	Basic fluid	*nf*	Nanofluid
*eff*	Effective	*s*	Solid
*f*	Fluid		
