# Supplementary material for: Heat Transfer and Hydrodynamic Properties Using Different Metal-Oxide Nanostructures in Horizontal Concentric Annular Tube: An Optimization Study
Source: Nanomaterials (Basel). 2021 Jul 31;11(8):1979. doi: 10.3390/nano11081979 (PMC8400204; doi:10.3390/nano11081979)
Supplement: Supplementary file 1 [file nanomaterials-11-01979-s001.zip › nanomaterials-1299746-supplementary.pdf]

Supplementary Materials

Article

Heat Transfer and Hydrodynamic Properties Using Different Metal-Oxide Nanostructures in Horizontal Concentric Annular Tube: An Optimization Study

Omer A. Alawi [1], Ali H. Abdelrazek [2], Mohammed Suleman Aldlemy [3], Waqar Ahmed [4,5], Omar A. Hussein [6], Sukaina Tuama Ghafel [7], Khaled Mohamed Khedher [8,9], Miklas Scholz [10,11,12,13,*] and Zaher Mundher Yaseen [14,15,*]

1 Department of Thermofluids, School of Mechanical Engineering, Universiti Teknologi Malaysia,
Skudai 81310, Johor Bahru, Malaysia; omeralawi@utm.my
2 Department of Mechanical Engineering, University of Malaya, Kuala Lumpur 50603, Malaysia;
ali_hassan80@siswa.um.edu.my
3 Department of Mechanical Engineering, Collage of Mechanical Engineering Technology, Benghazi, Libya; maldlemy@ceb.edu.ly
4 Malaysia-Japan International Institute of Technology (MJIIT), Kuala Lumpur 54100, Jalan Sultan Yahya Petra, Kuala Lumpur, Malaysia; waqarum.ah@gmail.com
5 Institute for Advanced Studies, University of Malaya, Kuala Lumpur 50603, Malaysia
6 Department of Mechanical Engineering, College of Engineering-Alsharkat, Tikrit University, Tikrit 34005, Iraq; omar-assi81@tu.edu.iq
7 Optical Department, Al-Ayen University, Nasiriyah 64001, Iraq; tuamasukaina88@alayen.edu.iq
8 Department of Civil Engineering, College of Engineering, King Khalid University, Abha 61421, Saudi Arabia; kkhedher@kku.edu.sa
9 Department of Civil Engineering, High Institute of Technological Studies, Mrezgua University Campus, Nabeul 8000, Tunisia
10 Division of Water Resources Engineering, Faculty of Engineering, Lund University, P.O. Box 118,
221 00 Lund, Sweden
11 Department of Civil Engineering Science, School of Civil Engineering and the Built Environment,
University of Johannesburg, Kingsway Campus, P.O. Box 524, Aukland Park 2006, Johannesburg 2092, South Africa
12 Department of Town Planning, Engineering Networks and Systems, South Ural State University (National Research University), 76, Lenin prospekt, 454080 Chelyabinsk, Russia
13 Institute of Environmental Engineering, Wroclaw University of Environmental and Life Sciences,
ul. Norwida 25, 50-375 Wrocław, Poland
14 New era and development in civil engineering research group, Scientific Research Center,
Al-Ayen University, Thi-Qar 64001, Iraq
15 College of Creative Design, Asia University, Taichung City, Taiwan
* Correspondence: miklas.scholz@tvrl.lth.se (M.S.); yaseen@alayen.edu.iq (Z.M.Y.)

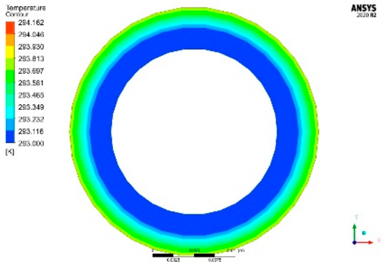

Al_2O_3-Nanoblades

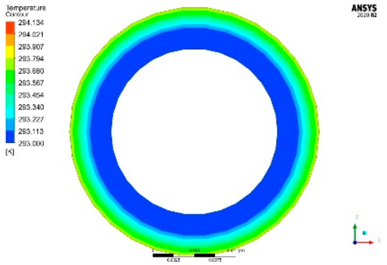

Al_2O_3-Nanobricks

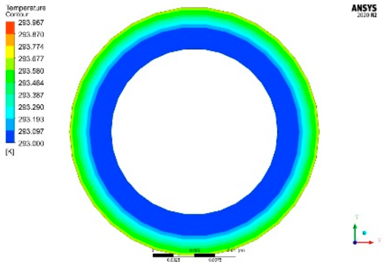

Al_2O_3-Nanocylinders

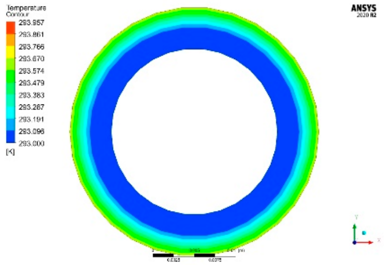

Al_2O_3-Nanoplatelets

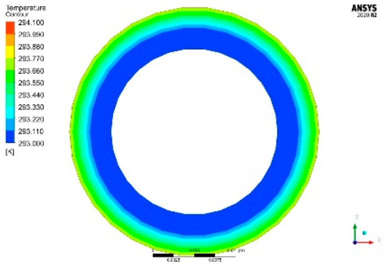

Al_2O_3-Nanospheres

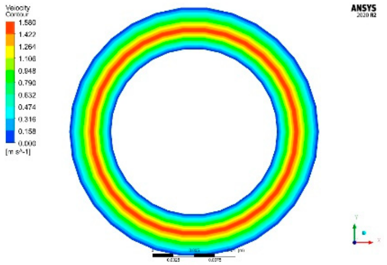

Al_2O_3-Nanoblades

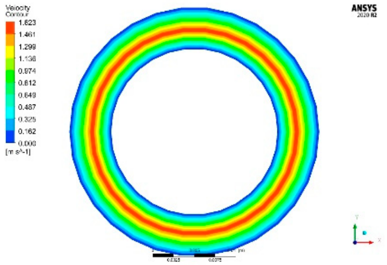

Al_2O_3-Nanobricks

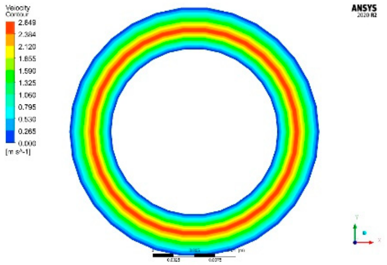

Al_2O_3-Nanocylinders

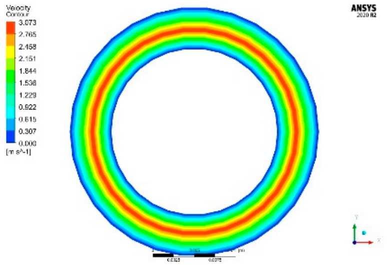

Al_2O_3-Nanoplatelets

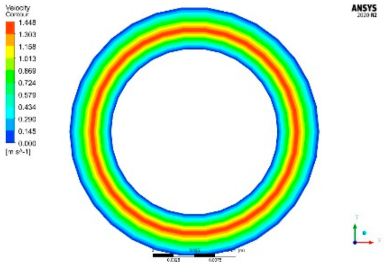

Al_2O_3-Nanospheres

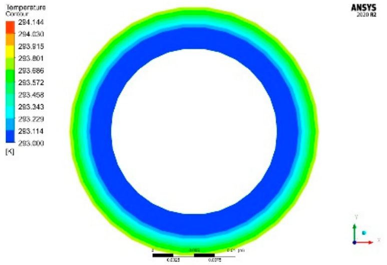

CuO-Nanoblades

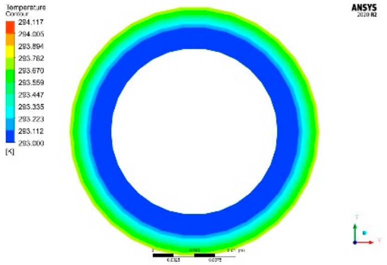

CuO-Nanobricks

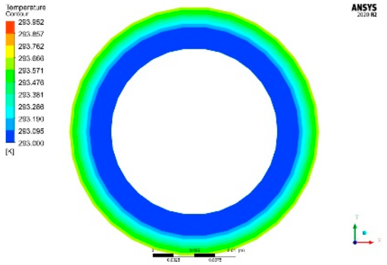

CuO-Nanocylinders

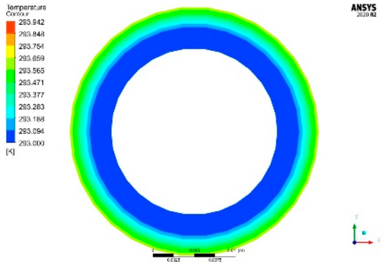

CuO-Nanoplatelets

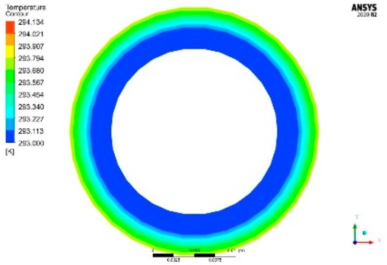

CuO-Nanospheres

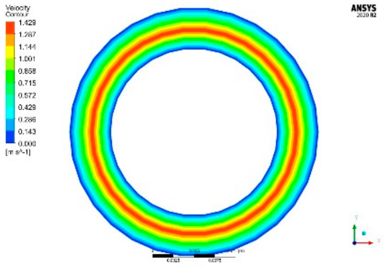

CuO-Nanoblades

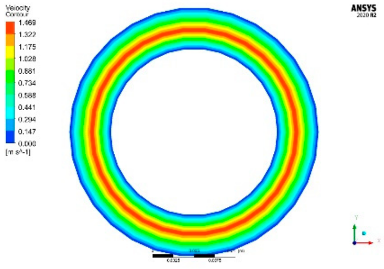

CuO-Nanobricks

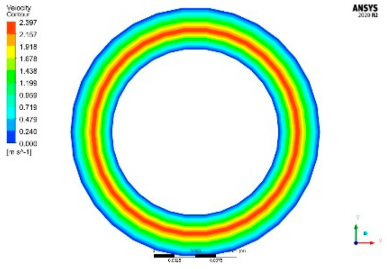

CuO-Nanocylinders

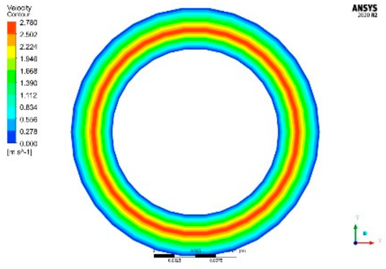

CuO-Nanoplatelets

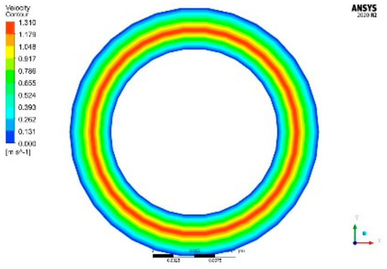

CuO-Nanospheres

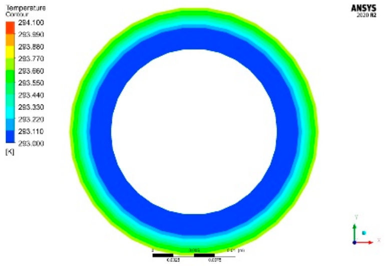

SiO_2-Nanoblades

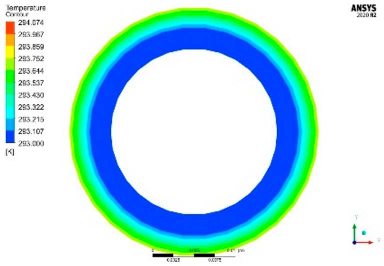

SiO_2-Nanobricks

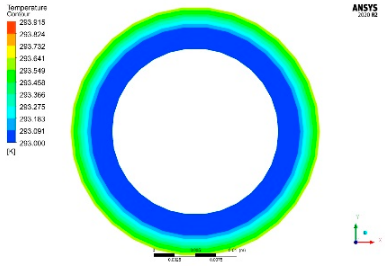

SiO_2-Nanocylinders

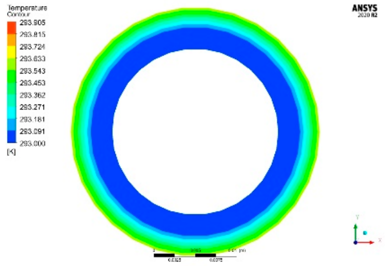

SiO_2-Nanoplatelets

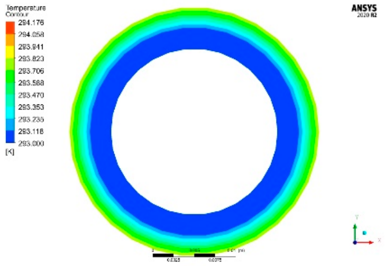

SiO_2-Nanospheres

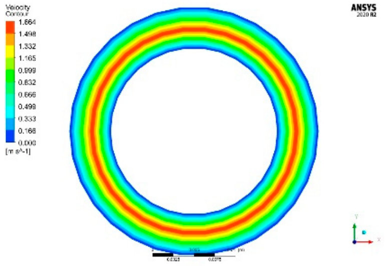

SiO_2-Nanoblades

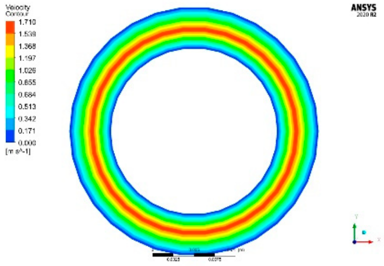

SiO_2-Nanobricks

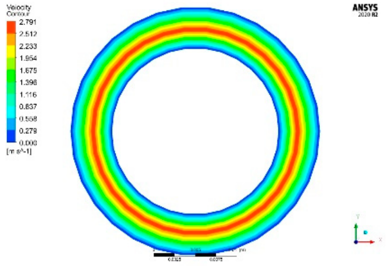

SiO_2-Nanocylinders

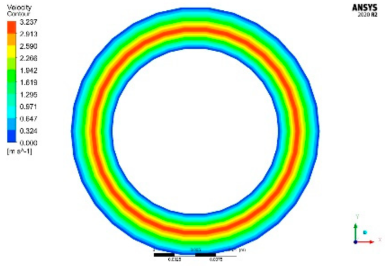

SiO_2-Nanoplatelets

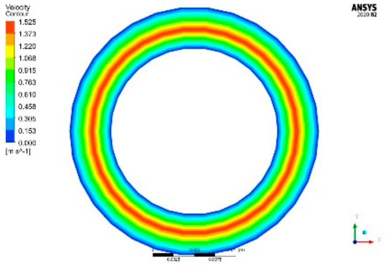

SiO_2-Nanospheres

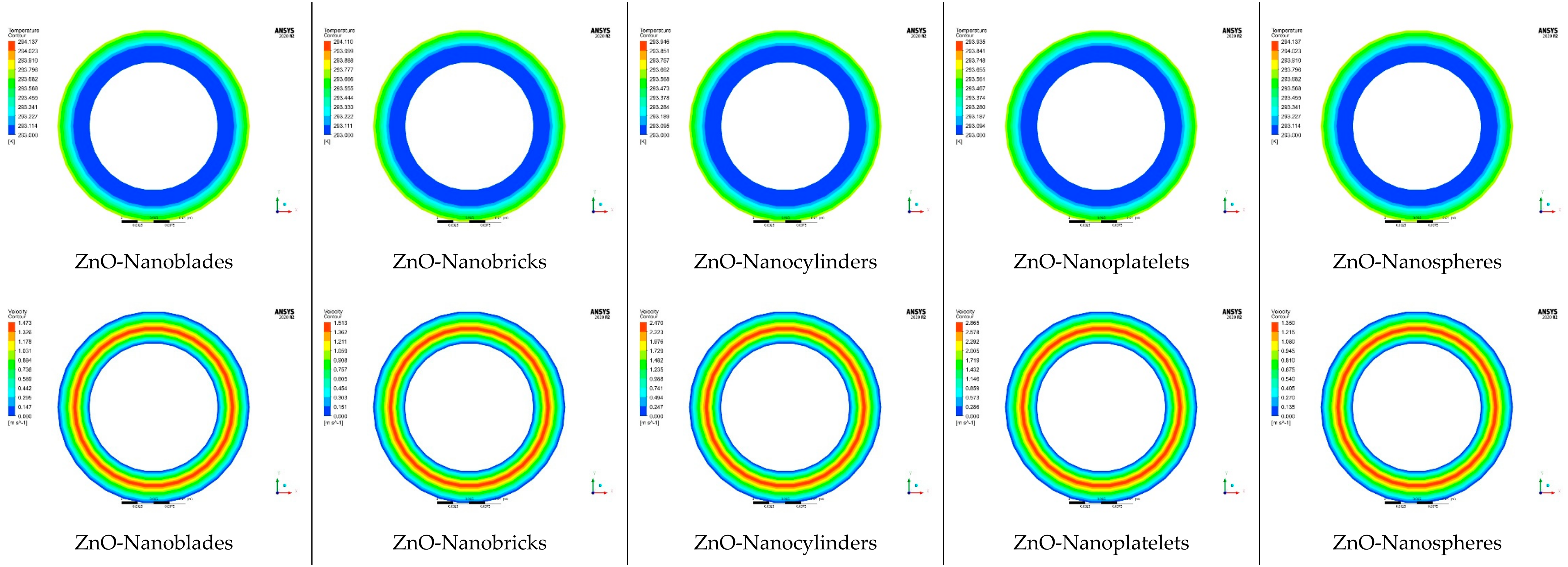

Figure S1. Temperature and velocity contours of different nanofluids and different nanoparticles shape under the conditions of 4 vol.%, 20 nm, 293 K, Re = 10,000 and 5000 W/m^2.

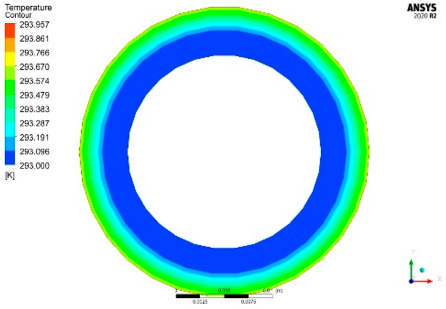

Al_2O_3-Nanoplatelets-HFR-0

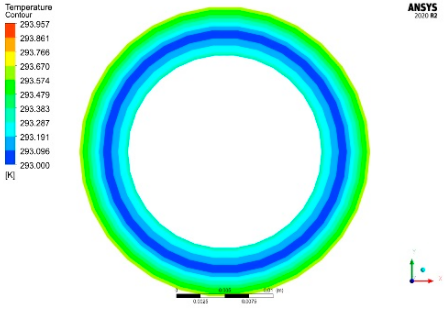

Al_2O_3-Nanoplatelets-HFR-0.5

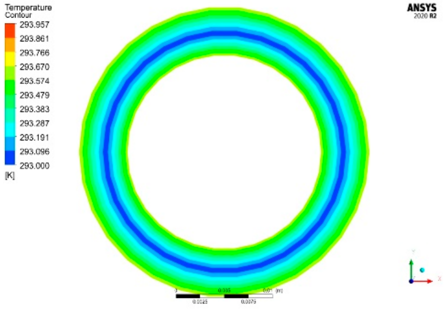

Al_2O_3-Nanoplatelets-HFR-1

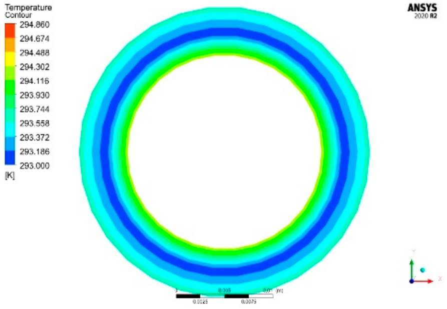

Al_2O_3-Nanoplatelets-HFR-2

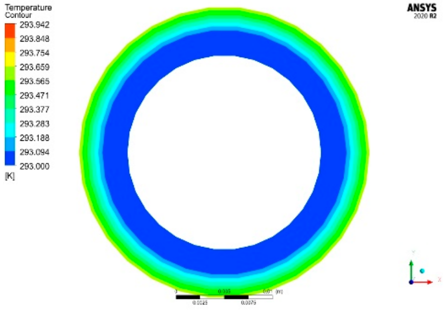

CuO-Nanoplatelets-HFR-0

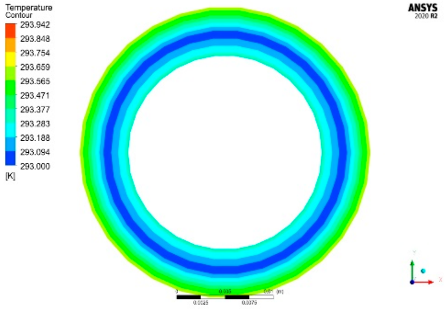

CuO-Nanoplatelets-HFR-0.5

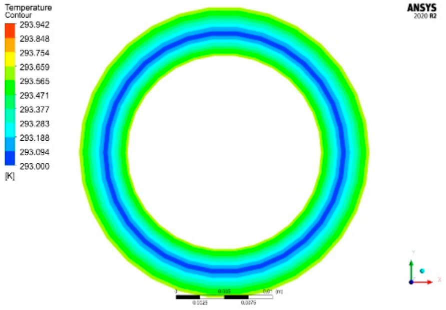

CuO-Nanoplatelets-HFR-1

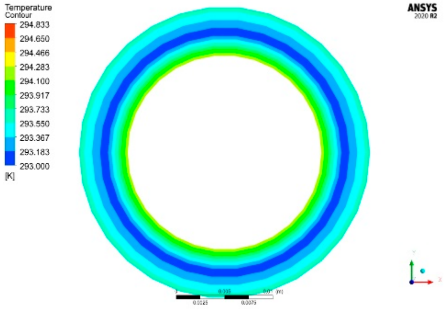

CuO-Nanoplatelets-HFR-2

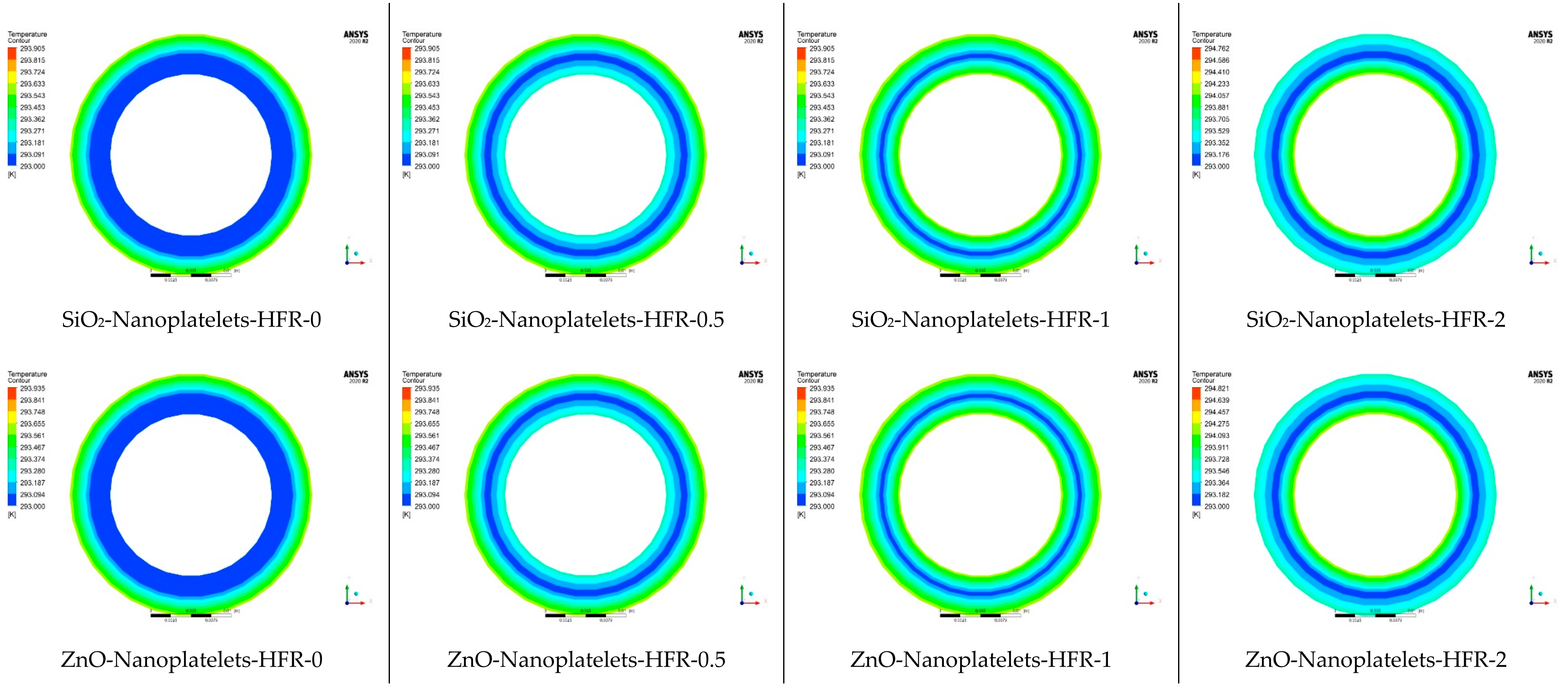

Figure S2. Temperature contours of different nanofluids and different heat flux ratios (HFR) under the conditions of 4 vol.%, 20 nm, 293 K, Re = 10,000 and 5000 W/m^2.

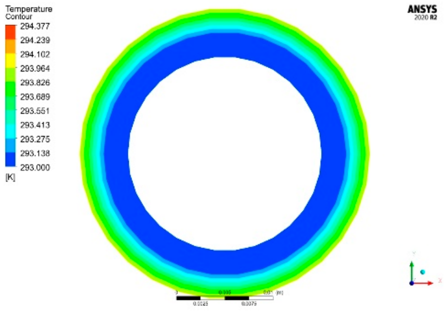

DW-T-AR@1.5

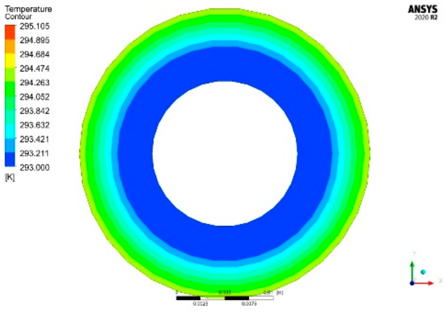

DW-T-AR@2

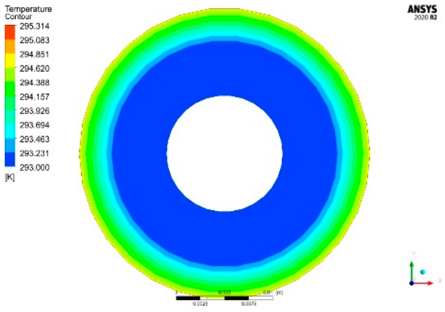

DW-T-AR@2.5

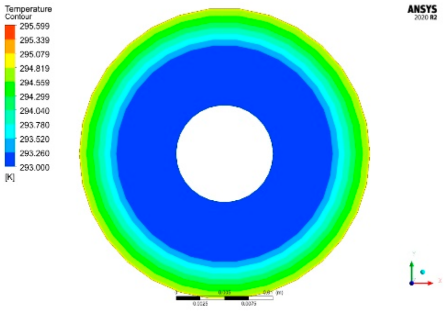

DW-T-AR@3

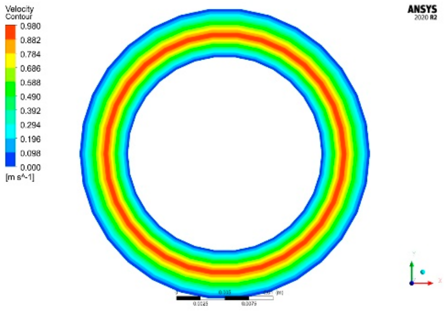

DW-V-AR@1.5

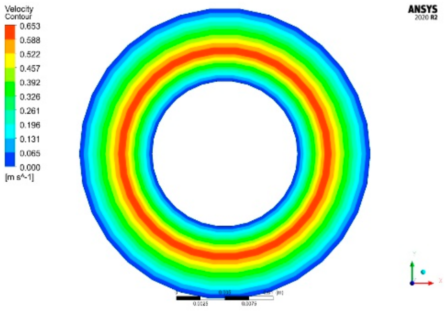

DW-V-AR@2

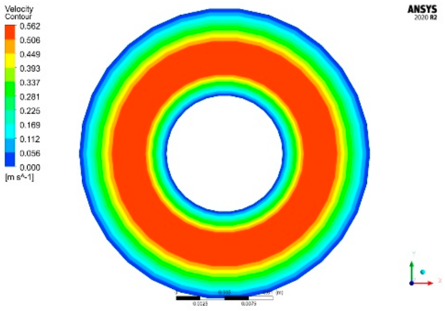

DW-V-AR@2.5

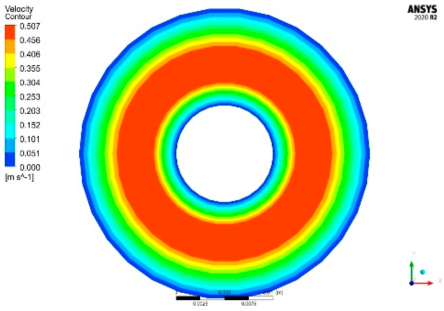

DW-V-AR@3

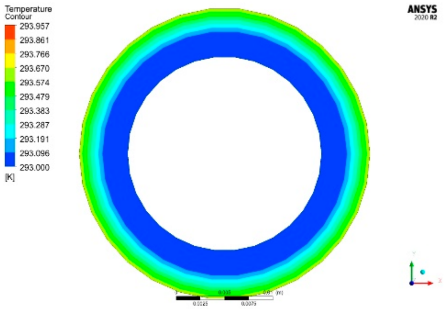

Al_2O_3-T-AR@1.5

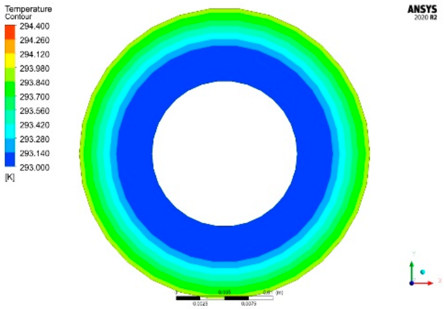

Al_2O_3-T-AR@2

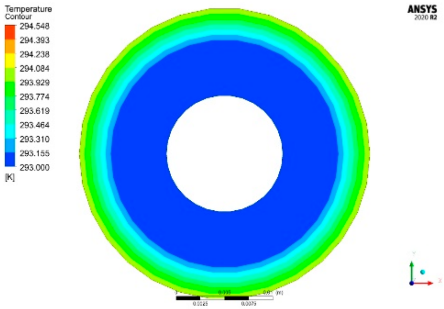

Al_2O_3-T-AR@2.5

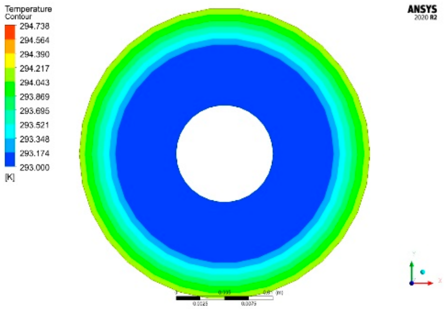

Al_2O_3-T-AR@3

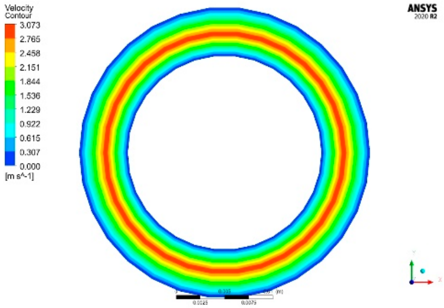

Al_2O_3-V-AR@1.5

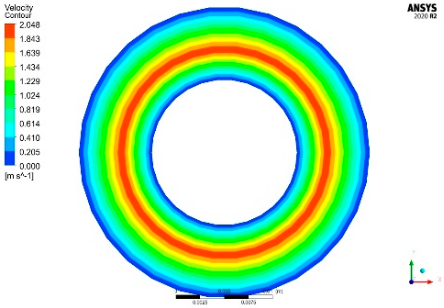

Al_2O_3-V-AR@2

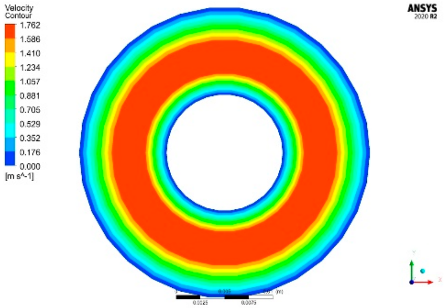

Al_2O_3-V-AR@2.5

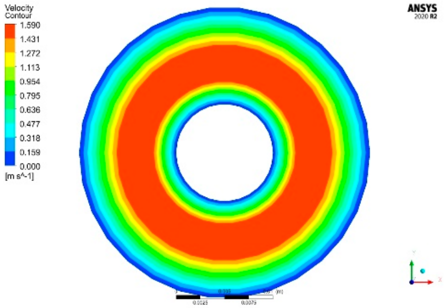

Al_2O_3-V-AR@3

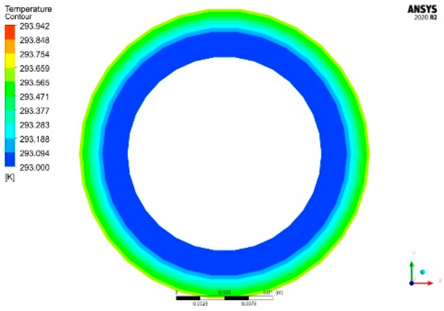

CuO-T-AR@1.5

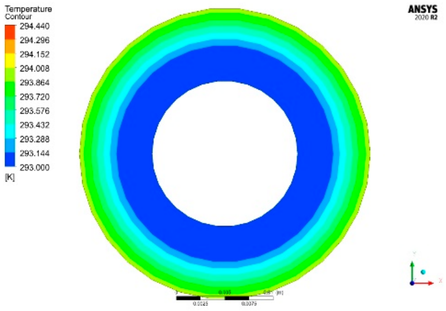

CuO-T-AR@2

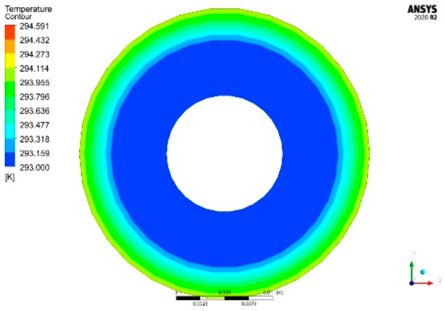

CuO-T-AR@2.5

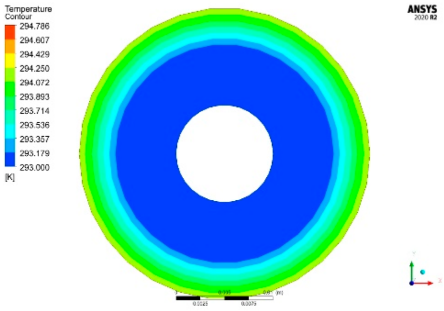

CuO-T-AR@3

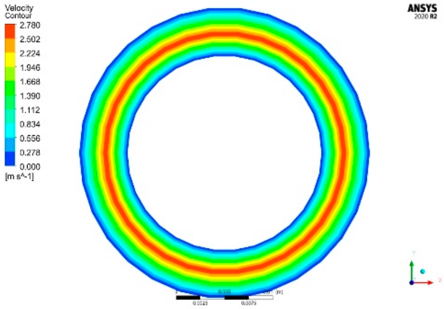

CuO-V-AR@1.5

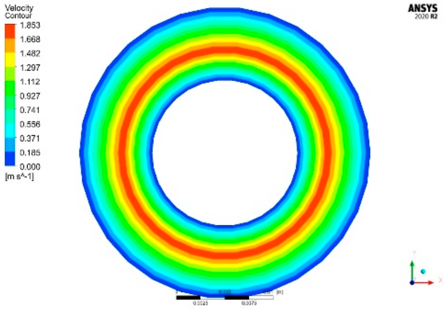

CuO-V-AR@2

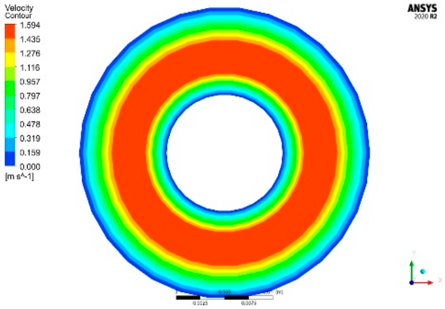

CuO-V-AR@2.5

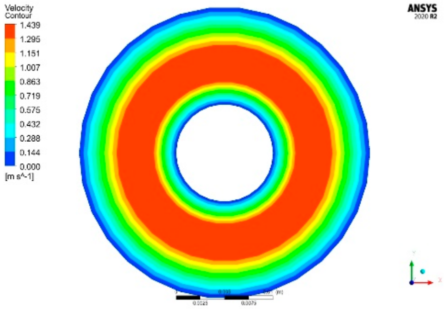

CuO-V-AR@3

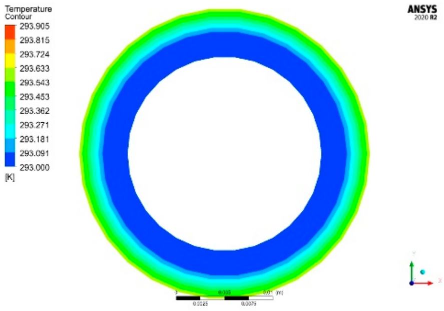

SiO_2-T-AR@1.5

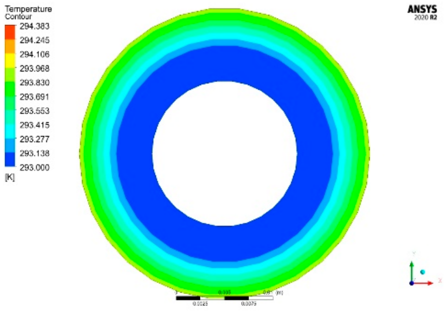

SiO_2-T-AR@2

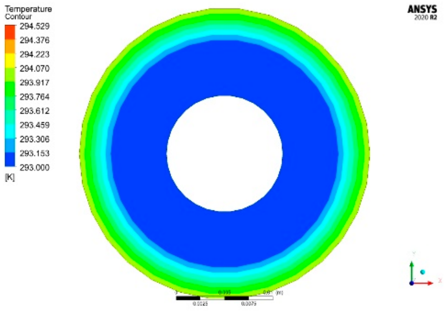

SiO_2-T-AR@2.5

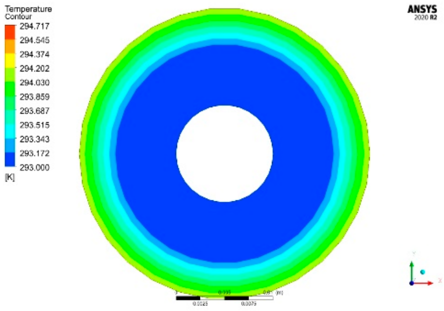

SiO_2-T-AR@3

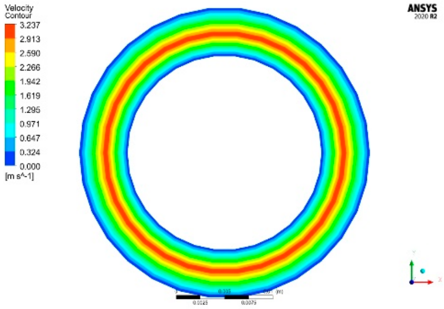

SiO_2-V-AR@1.5

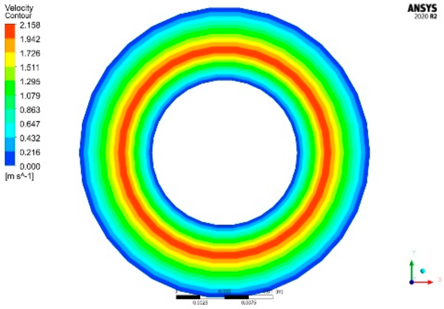

SiO_2-V-AR@2

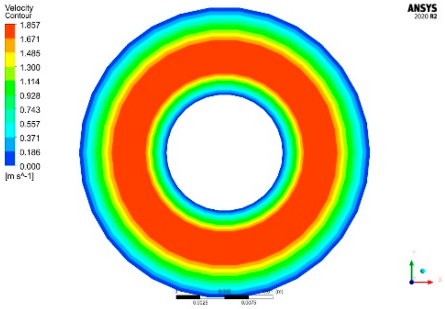

SiO_2-V-AR@2.5

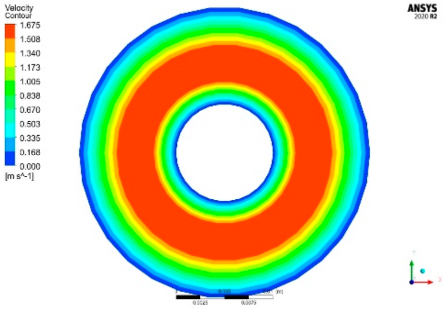

SiO_2-V-AR@3

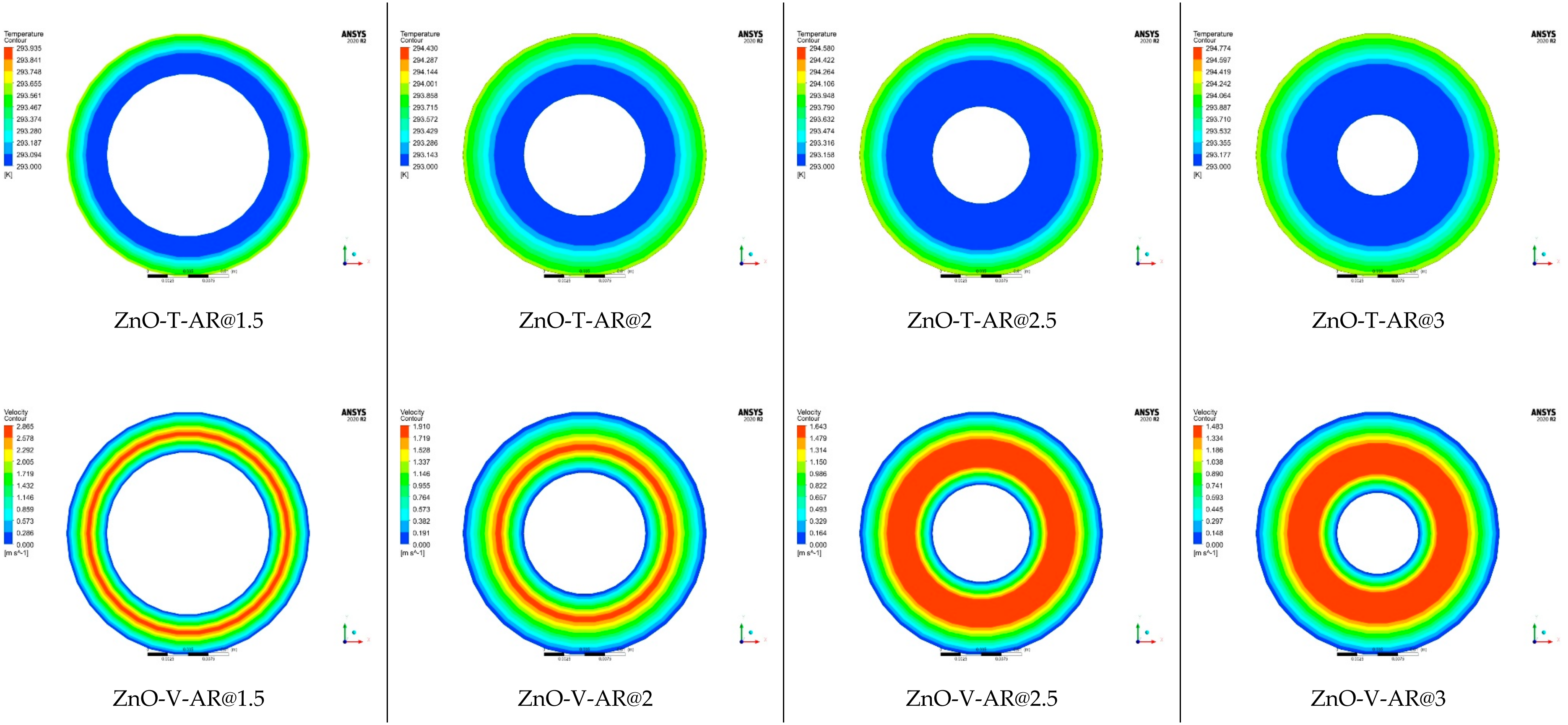

Figure S3. Temperature and velocity contours of DW and different nanofluid types (Al_2O_3, CuO, SiO_2, and ZnO) at 4 vol.%, 20 nm, 293 K and Re = 10,000.
